# The essential host genome for *Cryptosporidium* survival exposes metabolic dependencies that can be leveraged for treatment

**DOI:** 10.1016/j.cell.2025.07.001

**Published:** 2025-07-23

**Authors:** N. Bishara Marzook, Ok-Ryul Song, Lotta Baumgärtel, Netanya Bernitz, Tapoka T. Mkandawire, Lucy C. Watson, Vanessa Nunes, Scott Warchal, James I. MacRae, Michael Howell, Adam Sateriale

**Affiliations:** 1 The Cryptosporidiosis Laboratory, The Francis Crick Institute, London, UK; 2 High-throughput Screening Platform, The Francis Crick Institute, London, UK; 3 Metabolomics Science Technology Platform, The Francis Crick Institute, London, UK

## Abstract

*Cryptosporidium* is a leading cause of diarrheal disease, yet little is known regarding the infection cell biology of this intracellular intestinal parasite. To this end, we implemented an arrayed genome-wide CRISPR-Cas9 knockout screen to microscopically analyze multiple phenotypic features of a *Cryptosporidium* infection following individual host gene ablation. We discovered parasite survival within the host epithelial cell hinges on squalene, an intermediate metabolite in the host cholesterol biosynthesis pathway. A buildup of squalene within intestinal epithelial cells creates a reducing environment, making more reduced glutathione available for parasite uptake. Remarkably, the *Cryptosporidium* parasite has lost the ability to synthesize glutathione and has become dependent on this host import. This dependency can be leveraged for treatment with the abandoned drug lapaquistat, an inhibitor of host squalene synthase that shifts the redox environment, blocking *Cryptosporidium* growth *in vitro* and *in vivo*.

## Graphical abstract

**Graphical abstract:**
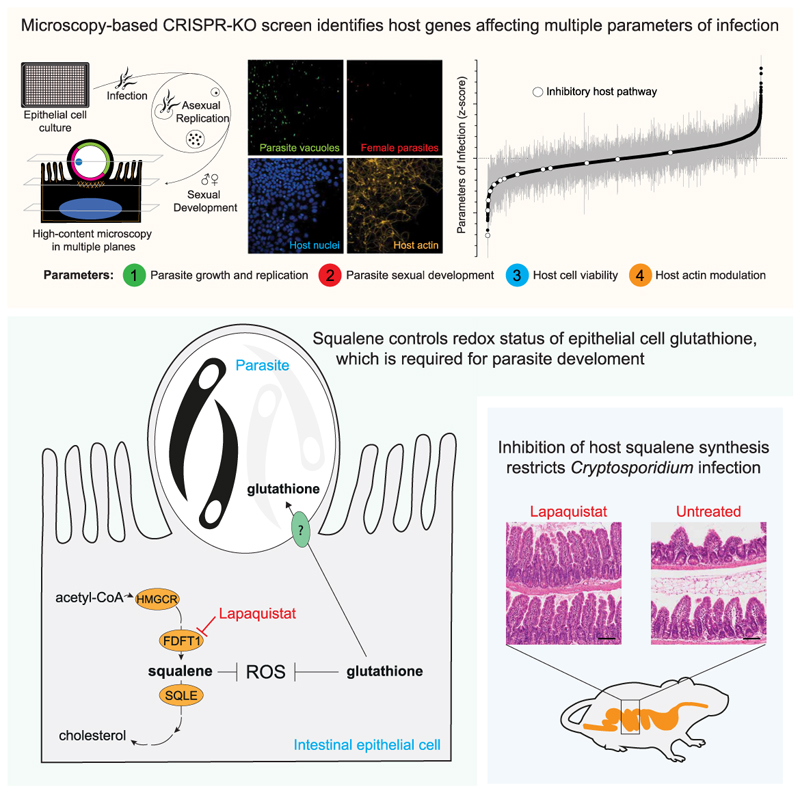

## Introduction

*Cryptosporidium* is an obligate intracellular parasite of the gut. Within the environment, the parasite exists in a dormant but infectious state known as an oocyst. Upon ingestion, this oocyst releases motile forms of the parasite that invade epithelial cells that line the intestine. Here, the parasite creates an apically localized vacuole, with a host actin-derived “pedestal” directly underneath. Inside this vacuole, the parasite will asexually replicate to exponentially infect neighboring epithelial cells. After multiple rounds of asexual replication, parasites enter the sexual stage of their life cycle, transforming into females (macrogamonts) or males (microgametes) that need to find each other to produce new infectious oocysts.^1,2^ Unlike other related apicomplexan parasites, such as *Plasmodium* and *Toxoplasma, Cryptosporidium* completes both asexual and sexual stages of its life cycle in a singular host, making it a unique system to better understand how host cell biology influences the parasite life cycle. Despite this, very little is known about the cell biology of infection of *Cryptosporidium*: what are the host factors that allow (or that are manipulated to allow) the parasite to invade, replicate, and survive within intestinal epithelial cells?

A growing appreciation of *Cryptosporidium*’s significant contribution to the burden of diarrheal-related deaths and disease in infants from lower-middle-income regions,^3–6^ the immunocompromised,^7^ and commercial livestock^8^ has recently spurred more active research into this parasite, particularly in the search for new therapeutics.^9^ This urgency is particularly pronounced as the only Food and Drug Administration (FDA)-approved drug for cryptosporidiosis, nitazoxanide, has proved ineffective in vulnerable populations.^10^ We reasoned that focusing on the parasite’s host dependencies would reveal un-explored insights into the cell biology of infection of this deadly parasite while also uncovering potentially novel therapeutic avenues to improve disease outcomes. To that end, we devised a microscopy-based arrayed CRISPR screen to systematically examine how protein-encoding human genes influence a *Cryptosporidium* infection. In previously described host cell-directed CRISPR screens, Cas9-expressing cells are first transduced in bulk with a pool of guide RNAs. This mixed population of host cells is then infected by the pathogen of interest, and surviving cells are sequenced to provide relative abundances of modified host cells before and after the assault.^11–14^ While powerful and broadly applicable, this approach only reveals one dimension of the infection, specifically, which genes impact host cell survival. In the arrayed system presented here, each host gene is ablated individually, and the subsequent pathogen infection is monitored by high-content imaging and automated image analysis pipelines. This results in a much richer dataset where the impact of host gene loss can be examined across many parameters of infection.

In our analysis, the parameters of *Cryptosporidium* infection that we primarily focused on were parasite growth, parasite sexual development, host cell viability, and host actin recruitment to parasite vacuoles. We show that while the loss of certain host genes and pathways increased parasite survival, others had an inhibitory effect. Curiously, perturbation of one host metabolic pathway—cholesterol biosynthesis—appeared to have opposing effects on parasite growth and development. On closer analysis, we discovered the ability of squalene, an intermediate metabolite in the cholesterol biosynthesis pathway, to control parasite survival and development. Although *Cryptosporidium* does not appear to import and utilize host squalene, we found the accumulation of this metabolite strongly influences reactive oxygen species (ROS) levels and glutathione (GSH) stores within intestinal epithelial cells. Further, the *Cryptosporidium* parasite appears to have lost the ability to synthesize its own GSH, becoming reliant on the stores within the infected host cell. This has created a host-targeted Achilles heel that we demonstrate can be exploited for treatment.

## Results

### A robust arrayed CRISPR-Cas9 screen for deconvoluting host-pathogen interactions

**Figure 1:**
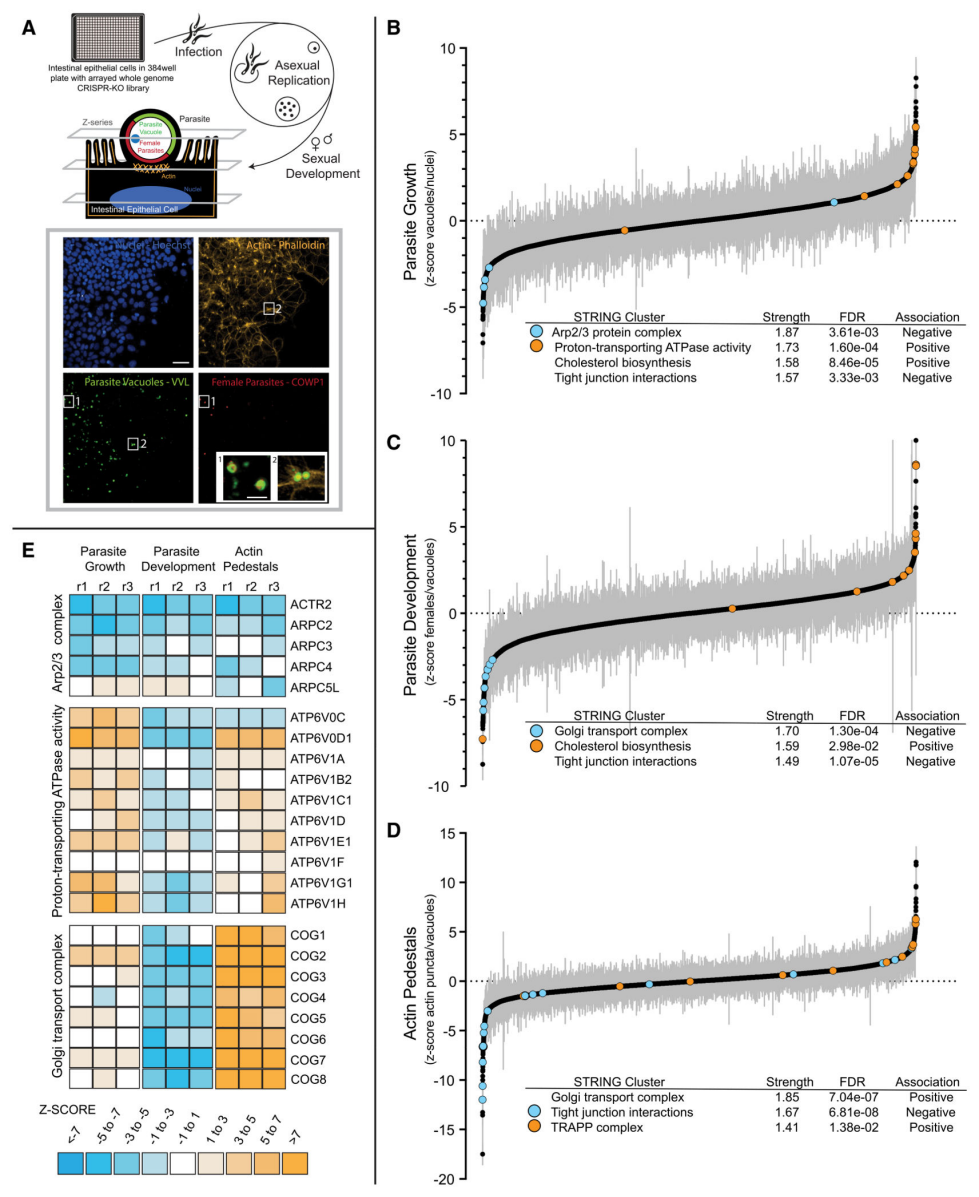
An arrayed full-genome CRISPR-Cas9 screen reveals host gene dependencies for multiple parameters of a *C. parvum* infection (A) Schematic of the arrayed full-genome CRISPR-Cas9 screen. HCT-8 cells expressing Cas9 were seeded into wells containing guide CRISPR RNAs (crRNAs). Each well contained four guides targeted to a single gene in the human genome. Three days later, cells were infected with *C. parvum*, followed by fixation 49 h post-infection. Cells were immunostained to detect all parasites, females, host actin, and nuclei and then imaged at three z stacks 3 μm apart to effectively capture host nuclei, actin pedestals beneath parasite vacuoles, and apically located intracellular *Cryptosporidium*. Scale bar, 20 μm; inset, 5 μm. (B–D) (B) Rank-ordering of all genes in the screen based on the effect of their knockout on parasite growth, (C) development of the female sexual stages, and (D) recruitment of host actin to “pedestals” beneath the parasite vacuole. Rank-ordering was based on median *Z* scores of three replicates per gene. (E) Representative examples of groups of genes in the same protein complex or pathway with significant *Z* scores in at least one parameter. *Z* scores for each replicate in each parameter are depicted as a heatmap.

*In vitro, Cryptosporidium* completes multiple asexual replication cycles and then commits to sexual development over a 48-h period. This developmental trajectory from asexual to sexual stages is thought to be necessary for the completion of this parasite’s life cycle.^2,15^ To map *Cryptosporidium*’s interactions with, and dependencies on, its host cell over this period, we devised a microscopy-based arrayed full-genome CRISPR screen to enable us to analyze several parameters of infection during this complex life cycle. For a detailed description of how we designed and conducted the screen, please see the STAR Methods section. Briefly, intestine epithelial cells (HCT-8) expressing Cas9 were seeded into clear-bottomed 384-well plates, where each well contained four guide RNAs targeting a single host gene. This was done for more than 18,000 human genes, covering virtually all human protein-coding genes in triplicate. Cells were then infected with *C. parvum* parasites and fixed 49 h post-infection (Figure 1A). Plates were prepared for immunofluorescence microscopy with fluorophores marking parasite vacuoles, female parasites, host actin, and host nuclei (Figure S1E). Each plate was subjected to high-content confocal microscopy to extract several image features from each channel for each well (see Table S1 for a detailed breakdown of image analysis steps). Therefore, for every host gene knocked out, we obtained an image-based multi-parametric readout of how this knockout (KO) affected multiple features of a *Cryptosporidium* infection (see Table S2 for a full list).

To make the best use of this data in the context of a *Cryptosporidium* infection, we narrowed our primary analyses to four infection hallmarks: (1) parasite growth (a ratio of the number of parasite vacuoles/number of host nuclei), (2) parasite development (a ratio of female parasites to total parasites), (3) parasite vacuoles with an associated actin pedestal, and (4) host cell viability (number of host nuclei). When we rank-ordered host genes by their median robust *Z* scores (measure of deviations from the median), we found a wide range of genes for which Cas9-assisted KO either reduced or promoted parasite growth, parasite development, or actin recruitment to parasite vacuoles (Figures 1B–1D). Under normal *in vitro* infection conditions, the percentage of parasite vacuoles with detected actin pedestals slightly increases with the multiplicity of parasite infection (Figure S1G), while the percentage of female parasites in the population at 49 h post-infection (hpi) remains constant even as the amount of infection increases (Figure S1G). This is in line with previous observations that *Cryptosporidium* sexual commitment is likely independent of parasite density.^2^

Next, we performed functional clustering analysis (via STRING v11.0b) of genes that produced a significant *Z* score in at least one parameter.^16^ Similar to transcriptomic results from genetic perturbation screens such as Perturb-seq,^17^ we noticed that groups of genes whose proteins exist in complexes or function within the same biological pathways often produced the same phenotype across multiple infection parameters (Figure 1E). For example, loss of six out of the seven members of the actin-nucleating actin-related proteins 2/3 (Arp2/3) complex, required for creation of branched filamentous actin (F-actin) filaments, reduced parasite numbers, development to female stages, and the formation of actin pedestals. Similarly, the loss of all 8 genes that function in the conserved oligomeric Golgi (COG) complex, which functions in intracellular transport, led to a uniform reduction in parasite development and an increase in actin pedestal formation during infection. Looking beyond groups of genes to the level of infection parameters, we found interesting correlations emerging between some of our selected image-based phenotypes (Figure S1K). For example, there was a significant positive correlation (r^2^ = 0.66) between host cell viability and parasite sexual development, yet no correlation between host cell viability and parasite growth (r^2^ = −0.12). This suggests that progression to sexual stages is more reliant on the health of the host cell than asexual replication.

### *Cryptosporidium* growth and development hinges on a metabolic tipping point in the host cholesterol pathway

**Figure 2:**
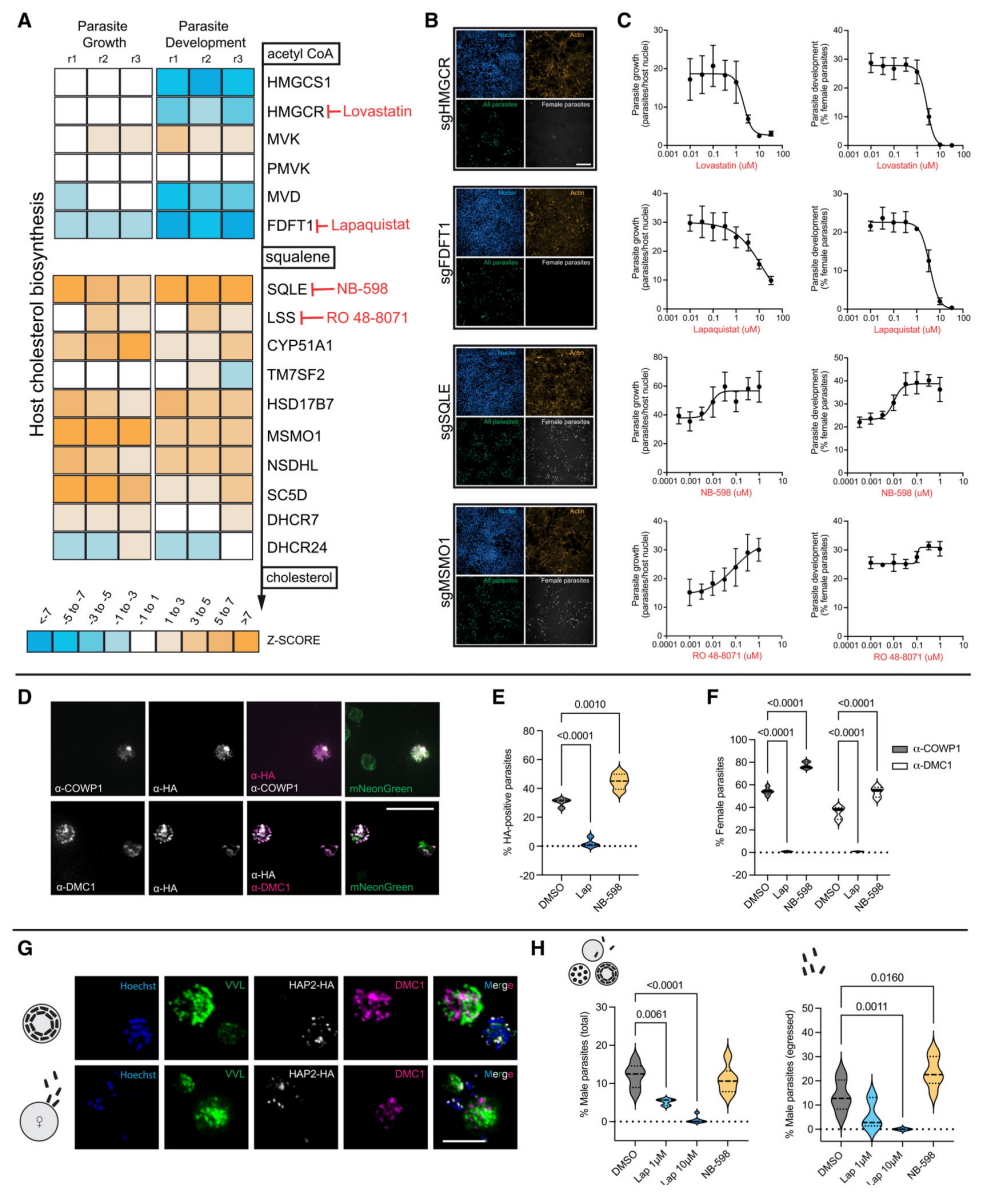
*Cryptosporidium* replication and sexual stage development hinge on a tipping point in the host cholesterol pathway (A) Heatmap of *Z* scores displaying the effect of knocking out genes in the host cholesterol biosynthesis pathway on parasite growth and sexual development, showing a shift at the midway point between *FDFT1* and *SQLE. Z* scores for all three replicates per gene are shown. (B) Representative images from the microscopy-based CRISPR screen for indicated genes in the cholesterol biosynthesis pathway showing a loss (for *HMGCR* and *FDFT1*) and increase (for *SQLE* and *MSMO1*) in total parasite numbers and females. Scale bar, 100 μm. (C) Dose-response curves using chemical inhibitors of indicated genes in (A), showing agreement with gene KO data in their effects on parasite replication (left) and female development (right). Parasite growth is calculated as the number of parasites per host nuclei expressed as a percentage, and female development, represented as the percentage of females in the total parasite population, is determined by anti-COWP1 staining. Data shown are the mean and standard deviation (SD) of 6 culture wells per condition and represent one of three experiments each for lovastatin, lapaquistat, and NB-598 and two experiments for RO 48-8071. (D) Immunofluorescence images of HCT-8 cells infected with transgenic *Cryptosporidium* parasites expressing mNeonGreen and COWP1 endogenously tagged with HA (expressed only in females). Anti-HA antibody staining agrees with the anti-COWP1 antibody used in the screen (top row) and anti-DMC1, another female-specific antibody (bottom row). Scale bar, 5 μm. (E) Treatment of HCT-8 cells infected with COWP1-HA-expressing parasites with lapaquistat (10 μM) or NB-598 (0.5 μM), respectively, reduces and increases the percentage of HA-expressing parasites 49 hpi. Data shown for 4 culture wells per condition. (F) Female parasite development in HCT-8 cells treated with 10 μM lapaquistat or 0.5 μM NB-598 was identified using either anti-COWP1 or anti-DMC1 antibody staining. The percentage of parasites that were DMC1-positive is slightly lower than COWP1-positive parasites. This is likely because DMC1 is transcribed later than COWP1 during female development; hence, only females that are further along in their development would be DMC1 positive.^18^ Data shown for 6 culture wells per condition. (G) Immunofluorescence images of HCT-8 cells infected with transgenic *Cryptosporidium* parasites expressing HAP2 endogenously tagged with HA (expressed only in males). Anti-HA staining identifies bullet-shaped male microgametes at the 16-nuclei stage (top row) and egressing males finding DMC1-positive females (bottom row). Scale bar, 2 μm. (H) The percentage of total male parasites (left) and egressed male parasites (right) in HCT-8 cells with indicated drug treatments. Data shown for at least 3 culture wells per condition from two independent experiments. Violin plots depict the median value at the heavy dashed line and the lower and upper quartiles at the lighter dashed lines. *p* values calculated using one-way ANOVA for (E) and (H) and two-way ANOVA for (F) using Dunnett’s multiple comparisons test. See also Figures S2 and S3.

*Cryptosporidium* lacks the ability to synthesize cholesterol and is thought to be reliant on the host to provide this essential metabolite. Therefore, one would predict that modulating this pathway would affect parasite growth and development. However, we were surprised to find that genes from the host cholesterol biosynthesis pathway were represented on either side of the *Cryptosporidium* growth and development spectra (Figures 1B and 1C). While the loss of some genes in this pathway inhibited parasite growth and sexual development, the loss of others enhanced these parameters. When we plotted the *Z* scores for each gene in the pathway going from acetyl-coenzyme A (CoA) to cholesterol, it revealed a midway point at which gene loss flipped from being inhibitory to parasite growth and development to promoting it (Figures 2A and 2B). This tipping point occurred between farnesyl-diphosphate farnesyltransferase 1 (*FDFT1*), whose loss reduced *Cryptosporidium* growth and development of females, and squalene epoxidase (*SQLE*), the loss of which enhanced growth and the percentage of females. For a more thorough breakdown of enzymes in the host cholesterol synthesis pathway, the intermediate metabolites they produce, and how their loss affected our different infection parameters, please see Figure S2.

A previous drug repurposing screen found statins, which act on 3-hydroxy-3-methylglutaryl coenzyme A (HMG-CoA) reductase (HMGCR), to be a reliable host target for reducing *Cryptosporidium* growth *in vitro*,^19^ so it was encouraging to see *HMGCR* coming up as a hit in our screen. We repeated this experiment using the HMGCR inhibitor lovastatin, and we further validated our screen results by using commercially available drugs targeting enzymes on either side of the *Cryptosporidium* growth divide (Figure 2C). This revealed that lapaquistat, which targets FDFT1, reduced parasite growth and development to females in a dose-dependent manner. NB-598, which inhibits host SQLE, recapitulated the *SQLE*-knockout (SQLE-KO) phenotype of enhancing growth and development, as did Ro 48–8071, which targets lanosterol synthase (LSS). Next, we were interested in which stage of infection was affected by our inhibitors. After invasion of host cells by individual parasites, they undergo many rounds of nuclear doubling within their apical vacuoles to progress from single nuclei (1n) to 2n, 4n, and finally 8n parasites. At this stage, these 8 infectious forms develop and leave their vacuoles to invade neighboring cells, repeating the asexual replication process a few more times before progression to male and female sexual stages at 36 h post-infection. Inhibitors could be acting at any step along this route. We found that pretreatment of host cells with lapaquistat or NB-598 did not affect parasite invasion into host cells (Figure S3A). While lapaquistat did not affect the overall proportions of different parasite life stages at 24 hpi, it significantly reduced the numbers of 1n and 8n parasites seen per field of view in a dose-dependent manner (Figures S3B and S3C). At 50 hpi, as expected, no female parasites were present at higher concentrations of lapaquistat, while the percentage of early-stage 1n, 2n, and 4n parasites increased. Therefore, while the detrimental effect of lapaquistat on *Cryptosporidium* sexual stage development is most pronounced, the drug appears to also stall asexual parasite development prior to sexual stage commitment. As NB-598 targets SQLE, the enzyme immediately following the target of lapaquistat, we wondered whether we could dampen the parasite growth-enhancing action of NB-598 with lapaquistat. Indeed, we found that increasing concentrations of lapaquistat correspondingly reduced the positive effects of NB-598 on parasite growth (Figure S3D).

To further verify the influence of the host cholesterol biosynthesis pathway on parasite development, we created a transgenic parasite line where the endogenous female-specific gene *Cryptosporidium* oocyst wall protein 1 (*COWP1*) is C-terminally fused to a hemagglutinin (HA) tag, followed by constitutive expression of mNeonGreen (Figures S3E and S3F). COWP1 antibodies co-localized with antibodies targeting the HA tag (Figure 2D, top row), and lapaquistat treatment nearly eliminated the presence of HA-positive parasites (females), while NB-598 enhanced them (Figure 2E). Furthermore, another female-specific antibody for *Cryptosporidium* raised against the meiosis-specific marker DNA Meiotic Recombinase 1 (DMC1)^20^ was able to specifically label COWP1-HA-positive parasites (Figure 2D, bottom row). DMC1 staining of infected cell monolayers revealed a similar boost from NB-598 treatment, whereas female parasites were nearly undetectable following treatment with lapaquistat (Figure 2F).

Next, we wanted to know if male parasites were similarly affected by these inhibitors. We created a transgenic parasite line where the male-specific gene *HAP2*^1^ was fused to an HA tag (Figures 2G and S3G) and treated this line with either lapaquistat or NB-598. As with females, lapaquistat reduced overall male parasite development in a dose-dependent manner (Figure 2H, left). At higher concentrations of lapaquistat, no females or males were observed. While NB-598 did not increase the total percentage of males in the population, when we stratified males by development stage, we found that NB-598 enhanced egress of male parasites, which is the final stage of male development (Figure 2H, right). In summary, we found that the inhibition of two consecutive enzymes in the host cholesterol biosynthesis pathway can either reduce or increase parasite growth and their overall development to male and female sexual stages *in vitro*.

### Squalene accumulation in the host epithelial cell reduces ROS and enhances *Cryptosporidium* growth

**Figure 3:**
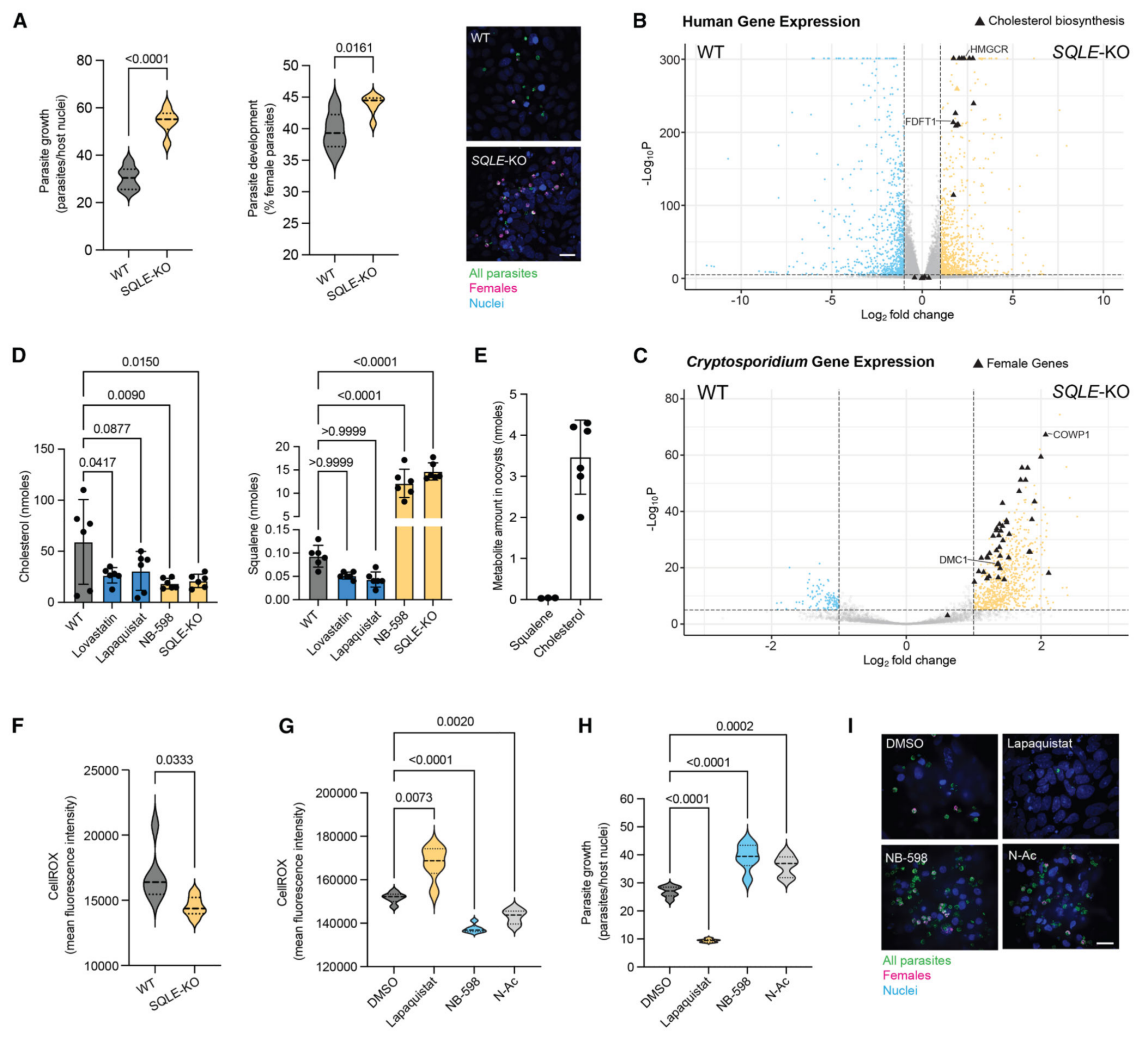
Squalene accumulation in epithelial cells reduces ROS and enhances *Cryptosporidium* growth (A) Parasite replication (left), female development (middle), and representative immunofluorescence images (left) of *Cryptosporidium* infection and presence of female parasites in parental or SQLE-KO HCT-8 cells. Data shown for 6 culture wells per condition, representative of four independent experiments. (B) Differentially expressed human genes and *Cryptosporidium* genes (C) In infected parental vs. SQLE-KO HCT-8 cells at 49 hpi, over 3 biological replicates. Horizontal dashed lines depict a *p* value cutoff of 1e–5, and vertical dashed lines are at a log_2_(fold change) of –1 and 1. (D) Quantification of cholesterol (left) and squalene (right) levels in HCT-8 cells with indicated drug treatments. (E) Levels of detectable cholesterol and squalene in *Cryptosporidium* oocysts. (F) Cellular ROS levels measured using CellROX in parental and SQLE-KO HCT-8 cells. Data shown for 6 culture wells per condition, representative of two independent experiments. (G) Cellular ROS levels measured using CellROX in infected HCT-8 cells 48 h after drug treatment. Data shown for 6 culture wells, representative of two independent experiments. (H) 48 h parasite growth in HCT-8 cells under indicated drug treatments calculated as the number of parasites per host nuclei expressed as a percentage. Data shown for 6 culture wells per condition, representative of three independent experiments. (I) Representative immunofluorescence images of WT *C. parvum* infection and presence of female parasites in HCT-8 cells with DMSO, lapaquistat (10 μM), NB-598 (0.5 μM), or N-Ac (10 mM). Scale bars, 10 μm. Violin plots depict the median value at the heavy dashed line and the lower and upper quartiles at the lighter dashed lines. *p* values calculated by t test with Welch’s correction for (A), Mann-Whitney test for (F), and one-way ANOVA with Dunnett’s multiple comparisons test for (D), (G), and (H). See also Figure S4.

To rule out any off-target effects of NB-598 treatment, we created an SQLE-KO HCT-8 cell line by Cas9-directed disruption of its genetic locus (Figures S4A and S4B). Loss of SQLE expression did not affect host cell viability (Figure S4C), and *Cryptosporidium* growth and development was indeed significantly enhanced in SQLE-KO cells compared with wild-type (WT) controls (Figure 3A). Differential expression analysis of transcriptomes from WT and SQLE-KO cells infected by *Cryptosporidium* revealed an upregulation of host cholesterol biosynthesis genes in SQLE-KO cells compared with WT (Figure 3B). Expression of cholesterol biosynthesis genes is tightly regulated by levels of cholesterol in the cell, sensed by sterol-responsive transcription factors such as the sterol regulatory element-binding proteins (SREBPs), which enhance transcription of cholesterol synthesis genes when cholesterol levels are low.^21,22^ SQLE is a key enzyme in the cholesterol synthesis pathway,^23^ and its loss would reduce cholesterol production, thereby activating transcription of cholesterol synthesis genes,^24^ which is what we found. *Cryptosporidium* parasites in SQLE-KO cells showed an upregulation of genes associated with late-stage female parasites compared with WT cells (Figure 3C), while the most upregulated parasite genes in WT cells by comparison were associated with late meronts, the final asexual life stage (Figure S4D). To pinpoint the consequences of changes to the host cholesterol biosynthesis pathway between WT and SQLE-KO cells, we next used a targeted metabolomics approach. Lovastatin treatment reduced host cholesterol levels as expected, as did both lapaquistat and NB-598 (Figure 3D, left), since they both target enzymes upstream of cholesterol production. Squalene is an intermediary metabolite in cholesterol biosynthesis, thus usually present at low levels. Treatment with either lovastatin or lapaquistat appeared to reduce squalene levels even further, as they both target enzymes upstream of squalene synthesis, while conversely, NB-598 treatment produced at least a 100-fold increase in squalene levels in host cells (Figure 3D, right). SQLE-KO cells had similarly elevated levels of squalene.

We considered that squalene could be taken up and either stored or used by the parasite, and this could be the cause of its improved growth under conditions of squalene accumulation in the host cell. We looked for squalene in oocysts, the environmentally released infectious forms of the parasite. Squalene was not detectable in oocysts, but cholesterol was (Figures 3E and S4E). Furthermore, our bioinformatic screens did not find any *Cryptosporidium* homologs for known genes that act on squalene. This suggested that while host cells supporting improved *Cryptosporidium* growth had greater levels of squalene, this metabolite does not appear to be imported or used by the parasite.

Squalene is a triterpene shown to be important for the survival of B-cell lymphomas by overcoming oxidative stress.^25^ We asked if squalene could be playing a similar role in epithelial cells. Cellular ROS levels were lower in SQLE-KO cells compared with WT HCT-8s (Figure 3F). Importantly, lapaquistat treatment of HCT-8 cells increased ROS within HCT-8 cells, while NB-598, which causes a squalene buildup, reduced ROS levels (Figure 3G). This ROS trend was negatively correlated with parasite growth, as seen previously. Interestingly, NB-598 reduced intracellular ROS to similar levels as our positive control, N-acetyl cysteine (N-Ac), which is commonly used as an antioxidant. Furthermore, treatment with N-Ac alone enhanced *Cryptosporidium* growth in HCT-8 cells, comparable to NB-598 treatment (Figures 3H and 3I). We also tested a range of other antioxidants on their ability to boost *Cryptosporidium* infection, finding several, such as AD4 (a more membrane-permeable derivative of N-Ac), tocopherol, and ascorbic acid, as being able to enhance either *Cryptosporidium* growth, sexual development, or both (Figure S5A).

### Squalene regulates levels of reduced GSH in epithelial cells

**Figure 4:**
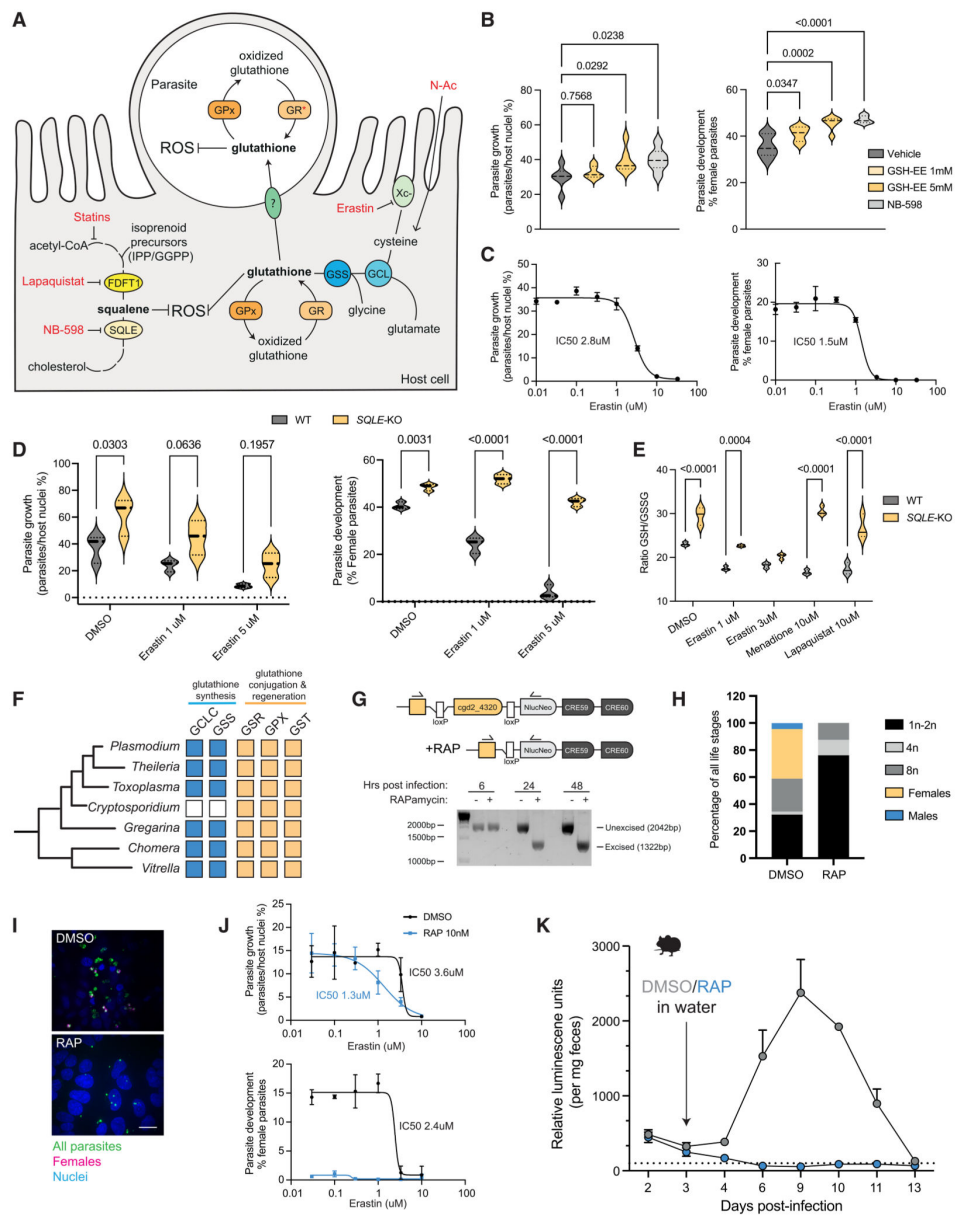
*Cryptosporidium* requires host glutathione to complete its life cycle (A) Schematic of proposed model for ROS reduction by squalene and N-Ac via increased availability of reduced host glutathione for uptake by *Cryptosporidium*. Points of action of various chemical inhibitors indicated. (B) Parasite replication (left) and female development (right) 49 hpi with glutathione ethyl ester (GSH-EE) and NB-598 treatment. Data shown for 6 culture wells per condition, representative of two independent experiments. (C) Dose-response curves for parasite replication (left) and female development (right) in HCT-8 cells with erastin treatment 49 hpi. Data shown are the mean and error for 3 culture wells per condition, representative of three independent experiments. (D) Dose-response curves for parasite replication (left) and female development (right) 49 hpi in either parental or SQLE-KO HCT-8 cells with erastin treatment. Data shown for three wells per condition, representative of two independent experiments. (E) Ratio of reduced to oxidized GSH in either parental or SQLE-KO HCT-8 cells 4 h after indicated drug treatments. Data shown for three wells per condition, representative of two independent experiments. (F) Presence of genes for GSH synthesis (mustard) or GSH recycling and conjugation (blue) in apicomplexan parasites and nearest relatives. (G) Schematic for the creation of transgenic *C. parvum* with *loxP* sites flanking the dimerization domain of its glutathione reductase gene and an inducible Cre recombinase (called GR-KO from here on; top). Addition of 10 nM rapamycin causes an excision of the *loxP*-flanked region of the parasite *GR* gene. Immunofluorescence images of HCT-8 cells infected with the inducible GR-KO parasites with or without rapamycin treatment (below). (H) Percentage of GR-KO parasites at indicated life stages in HCT-8 cells treated with either DMSO or 10 nM rapamycin 49 hpi. 90 parasites were counted for the DMSO condition, and 88 parasites were counted for the rapamycin condition. (I) Representative immunofluorescence images of inducible GR-KO parasite infection and presence of female parasites in HCT-8 cells with or without rapamycin (RAP) treatment. Scale bar, 10 μm. (J) Dose-response curves for replication (top) and female development (bottom) of GR-KO parasites in HCT-8 cells under erastin treatment with or without 10 nM rapamycin to induce loss of parasite *GR*. Data shown are the mean and error for three culture wells per condition. (K) Fecal nanoluciferase readings from *IFN*γ*R*^−/−^ mice infected with inducible GR-KO parasites over 2 weeks. This transgenic parasite line also carries a nanoluciferase (Nluc)-expressing gene so parasite burdens can be assessed in mice by measuring fecal Nluc readings. Mice were infected by oral gavage and then either given DMSO or rapamycin in their drinking water 3 days post-infection, and 2 mice per condition. Violin plots depict the median value at the heavy dashed line and the lower and upper quartiles at the lighter dashed lines. *p* values calculated by one-way ANOVA with Dunnett’s multiple comparisons test (B) and two-way ANOVA with Šídák’s multiple comparisons test (D) and (E). See also Figure S5.

An accumulation of the intermediary metabolite squalene was able to reduce cellular ROS and promote parasite growth (Figure 4A). The principal mechanism by which cells regulate ROS is via the ubiquitous antioxidant metabolite GSH. Could increased levels of GSH alone be beneficial to *Cryptosporidium*? We first tested this by supplementing our media with GSH ethyl ester (GSH-EE), a cell-permeable formulation of GSH. Increasing amounts of GSH-EE alone correspondingly increased parasite growth and enhanced the percentage of female parasites to similar levels as NB-598 (Figure 4B). Erastin is a small molecule that inhibits the cellular import of cystine, a key component of GSH, thus lowering the levels of intracellular GSH^26^ (Figure 4A). We found that erastin reduced parasite growth and development in a dose-dependent manner (Figure 4C). Crucially, it is effective at stopping parasite growth at concentrations that do not affect host cell viability (Figure S5B). Another inhibitor of the GSH synthesis pathway is buthionine sulfoximine (BSO), which targets gamma-glutamylcysteine synthase (GSS). BSO was similarly able to inhibit *Cryptosporidium* growth and sexual development (Figure S5C).

We next asked whether the pro-oxidative effects of erastin could be countered by the antioxidant effects of squalene. WT or SQLE-KO cells were infected with *Cryptosporidium* and treated with increasing amounts of erastin. While growth and sexual development in WT cells faltered early with lower levels of erastin treatment, parasites grown in SQLE-KO cells were much more resistant to erastin (Figure 4D). Modulators of host ROS via GSH have also been implicated in a specialized form of cell death mediated by lipid peroxidation known as ferroptosis.^27^ We tested several reported inducers of ferroptosis, such as the ferroptosis-inducing peroxide FINO2, RSL3, and auranofin (Figures S5D–S5F), and while parasite growth was inhibited, these compounds also greatly reduced host cell viability, confounding any possible interpretations of the results.

Finally, as a buildup of squalene was able to enhance parasite growth and development to similar levels as GSH-EE alone, we hypothesized that squalene may act by maintaining higher levels of reduced cellular GSH. To test this, we measured the ratio of reduced to oxidized GSH in WT and SQLE-KO cells with different inducers of ROS. SQLE-KO cells maintained significantly higher levels of reduced:oxidized GSH (GSH:GSSG) in response to various ROS inducers, including lapaquistat and known cellular GSH depleters erastin and menadione (Figure 4E). Thus, increased levels of squalene in epithelial cells can buffer against GSH oxidation in the event of ROS induction, thereby maintaining a higher cellular pool of reduced GSH that is available for *Cryptosporidium*.

### *Cryptosporidium* requires host GSH for completion of its life cycle

How might host GSH be beneficial to *Cryptosporidium*? Interestingly, unlike most other eukaryotes as well as related parasites in its apicomplexan phylum, *Cryptosporidium* does not possess the genes to synthesize its own GSH (Figure 4F). It does, however, possess the genes to recycle GSH from its oxidized to reduced states (predominantly expressed during asexual life stages),^18^ as well as glutathione S-transferases (GSTs), which use GSH to post-translationally modify other proteins (expressed during male and female sexual stages).^18^ Consequently, while it cannot make its own GSH, the possession of these GSH-utilizing genes implies *Cryptosporidium* uses this essential metabolite and must obtain GSH from its host cell.

To test the importance of GSH for parasite fitness, we chose to impair *Cryptosporidium*’s capacity to recycle oxidized GSH by targeting its single *glutathione reductase* (*GR*) gene (cgd2_4320) for controlled disruption using an inducible gene KO system (Figure 4G). Briefly, the presence of rapamycin brings two subunits of a Cre recombinase together, enabling excision of the *loxP*-flanked dimerization domain of the parasite’s *GR* gene. In the presence of rapamycin, the *loxP*-flanked domain is still intact 6 hpi but is completely excised 24 hpi (Figure 4G). Hence, in rapamycin-treated GR-KO parasites, we predict loss of GSH reductase gene activity would be complete by the parasite’s third asexual replication cycle and before its progression to sexual development. Rapamycin treatment of infected cells alone does not affect a WT *Cryptosporidium* infection *in vitro* (data not shown); however, the addition of rapamycin to an infection with this transgenic strain abolished the development of male and female parasites and skewed asexual parasites toward smaller, earlier stages of development (Figures 4H and 4I). While the overall numbers of parasites were not reduced, their asexual and sexual development trajectories were impacted (data not shown). We conclude that removing the parasite’s ability to recycle GSH stalls parasite growth and prevents sexual stage progression, similar to the effects of lapaquistat.

As erastin decreases host GSH levels, we tested the impact of increasing concentrations of erastin on the inducible GR-KO line. KO parasites were more sensitive to erastin, with their growth IC50 (half-maximal inhibitory concentration) reducing almost 3-fold (Figure 4J). As loss of GR alone completely prevents sexual stage development, no further reductions in female parasite numbers could be assessed with erastin treatment. Finally, we tested the consequences of GR loss on a *Cryptosporidium* infection in mice. We infected two cohorts of interferon (IFN)-γ--deficient mice with inducible GR-KO parasites and administered either rapamycin or DMSO in their drinking water 3 days post-infection. GR-KO parasites in mice treated with rapamycin failed to establish a productive infection, with parasite burdens dropping below the detectable threshold 3 days post-rapamycin administration (Figure 4K). Collectively, these data show that GSH, a metabolite that the parasite cannot synthesize *de novo*, is essential for *Cryptosporidium* survival *in vivo*.

### Lapaquistat restricts *Cryptosporidium* infection in mice

**Figure 5:**
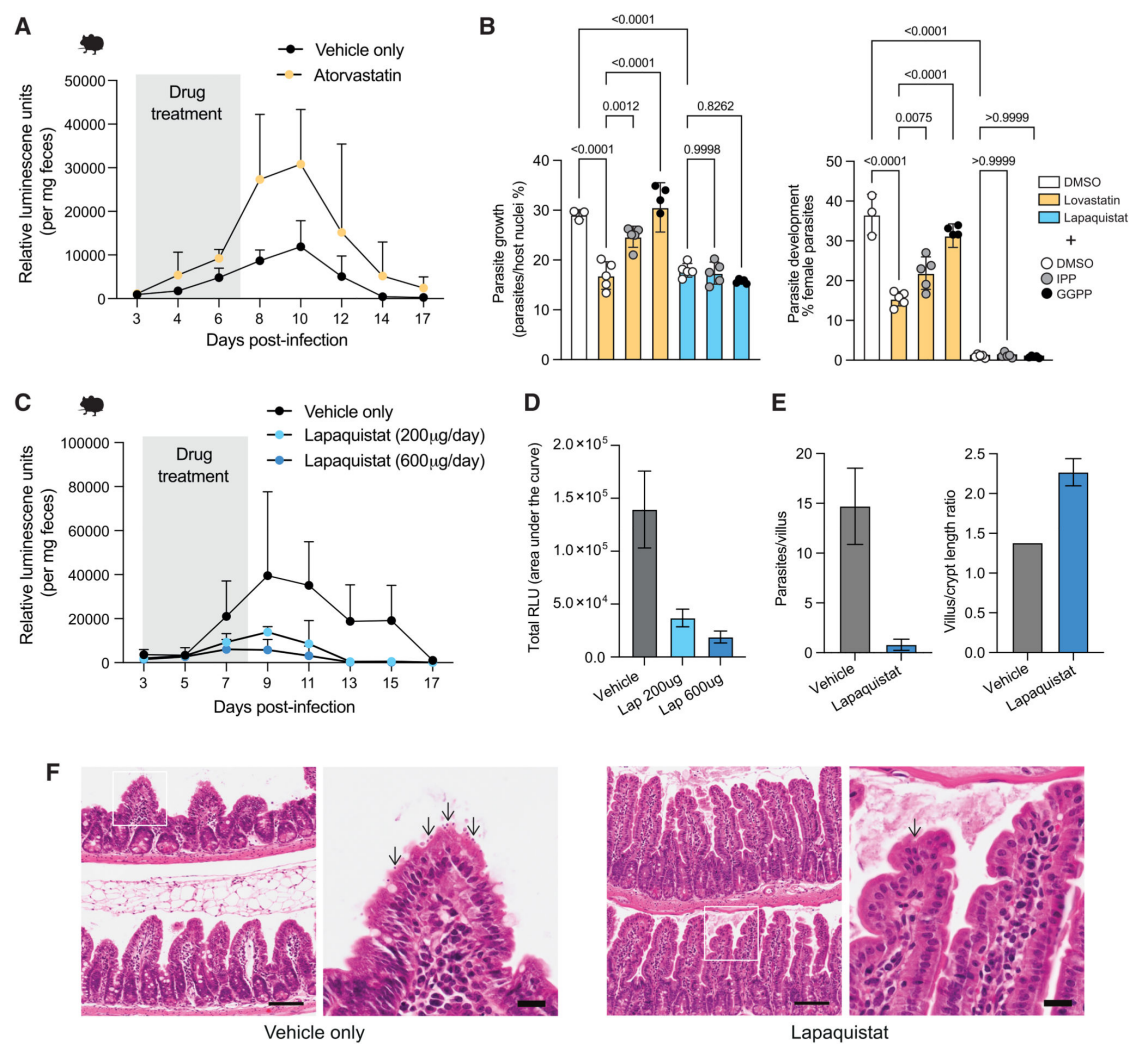
Lapaquistat restricts *Cryptosporidium* infection in mice (A) Fecal Nluc readings from *IFN*γ*R*^−/−^ mice infected with a mouse-adapted *C. parvum*-mCherry line expressing Nluc. Mice were infected and given atorvastatin (200 μg/mouse) or vehicle once daily by oral gavage on days 3–7 post-infection. 5 mice per condition, and 2 mice per condition were culled on day 10 for intestinal histopathology. Mean and SD are plotted. (B) Replication (left) or female development (right) of WT parasites infecting HCT-8 cells treated with either lovastatin or lapaquistat and additional isoprenoids as indicated. Data shown are the mean and SD for at least 3 culture wells per condition, representative of two independent experiments. *p* values calculated by two-way ANOVA using Tukey’s multiple comparisons test. (C) Fecal Nluc readings from *IFN*γ*R*^−/−^ mice infected with a mouse-adapted *C. parvum* line expressing Nluc. Mice were infected and given either a low (200 μg/mouse) or high (600 μg/mouse) dose of lapaquistat or vehicle only once daily by oral gavage on days 3–8 post-infection. 4 mice per condition, and one mouse in the vehicle-only cohort reached a humane endpoint on day 10. Mean and SD are plotted. (D) Areas under the curve for Nluc levels in (C). Mean and SEM are shown. (E) *C. parvum*-infected mice treated with either lapaquistat or vehicle were culled on day 10 post-infection, and ileal sections were examined by H&E staining to count the number of visible parasites per villus (left) and villus-crypt height ratios (right). 2 mice per condition. Mean and SD are plotted. (F) Representative images of the ileum from (E). Scale bars are 100 μm for the main images and 20 μm for the insets. See also Figure S5.

In light of our evidence that inhibiting host HMGCR either genetically or chemically during an infection was sufficient to restrict parasite infection in cell culture, we were curious as to whether statins, being readily commercially available and already FDA-approved for lowering cholesterol, would also work to reduce *Cryptosporidium* growth in mice. Surprisingly, we found that treatment of *IFN*γ*R*^−/−^ mice with atorvastatin did not reduce the parasite burden in mice (Figure 5A). Mammalian cholesterol biosynthesis begins with acetyl-CoA, but there are intermediary metabolites that can feed into this pathway, such as isoprenoid precursors (Figure 4A). It has been shown that treatment with the isoprenoid precursor isopentenyl pyrophosphate (IPP) partially blocks the inhibitory effect of statins on *Cryptosporidium* growth *in vitro*,^19^ and we arrived at a similar result. Inhibition of growth and parasite sexual stage development by statins was rescued by the exogenous addition of IPP or geranylgeranyl diphosphate (GGPP) (Figure 5B). By contrast, the inhibitory effect on growth and development of the parasite by lapaquistat, which inhibits FDFT1 downstream of isoprenoid entry in the cholesterol synthesis pathway, could not be rescued by the addition of isoprenoid precursors. This suggests that statins fail to limit parasite growth *in vivo* due to the presence of exogenous isoprenoids in the gut, which can bypass statin inhibition, which occurs early in the cholesterol synthesis pathway.

Unlike statins, the efficacy of lapaquistat *in vitro* correlated with *in vivo* treatment. Within a murine model of acute infection, we found that treatment of infected mice, using doses relative (by allometric scaling) to those previously trialed in human safety studies,^28^ significantly reduced parasite burden compared with vehicle-treated controls (Figure 5C). When vehicle- or lapaquistat-treated mice were culled at their infection peak, we noticed reduced numbers of parasites within the ileum of lapaquistat-treated mice (Figure 5D). Lapaquistat-treated mice also showed a decreased level of small intestinal damage that is commonly associated with *Cryptosporidium* infections, such as a decreased villus/crypt length ratio (Figures 5E and 5F). Because lapaquistat already has a considerable amount of clinical trial safety data for the treatment of hypercholesterolemia, it is a highly attractive candidate for repurposing as an anti-cryptosporidial drug.

## Discussion

A systematic interrogation of 18,466 protein-coding human genes and its effects on *Cryptosporidium* infection revealed unexpected insights into host and parasite cell biology, as well as a new host-directed therapeutic target for treatment of the deadly disease caused by this pathogen. We found FDFT1, the first enzyme representing commitment to cholesterol biosynthesis, to be critical for *Cryptosporidium* growth and sexual stage development. Treatment of infected mice with an inhibitor of FDFT1, lapaquistat, reduced the parasite burden and hallmarks of intestinal damage. Lapaquistat was initially developed as an alternative cholesterol-lowering drug to statins and progressed to several phase 3 clinical trials, including one where subjects were given low or high doses of the drug (equivalent to the low or high doses given to mice in our study), both of which effectively reduced plasma low-density lipoprotein (LDL) cholesterol.^28^ A small number of participants (two out of more than 5,000) given the highest drug dose developed mildly elevated liver enzymes over a dosage period exceeding 8–12 weeks, which ultimately halted its progress to the market. This effect is believed to be on-target, as liver-specific *FDFT1* KO mice exhibited a similar increase in liver enzymes and a buildup of isoprenoid precursors.^29^ Importantly, liver enzyme levels normalized in all patients of the lapaquistat study once treatment was halted, and no long-term effects were reported. Given our finding of lapaquistat’s success in restricting *Cryptosporidium* infection in immunocompromised mice over a much shorter dosage window in this study, we are hopeful for its repurposing as an anti-cryptosporidial therapeutic. This is urgently needed since the only other FDA-approved drug for *Cryptosporidium* treatment, nitazoxanide, does not work in vulnerable populations.^9,10^ Additionally, development of drug resistance is a huge barrier to fighting bacterial and parasitic diseases,^30–35^ which many pathogen-targeted drug discovery studies ignore. Host-directed therapies have a much lower probability of enabling the development of resistance and are being pursued as alternative strategies against other pathogens such as *Plasmodium*,^36^ *Salmonella*,^37^ and SARS-CoV-2.^12^ Despite lovastatin and lapaquistat exhibiting synergistic potential to curb *Cryptosporidium* infection *in vitro* (Figures S5G–S5I), the failure of statins alone in mice and the rescue of parasite growth by isoprenoids *in vitro* cautioned us against pursuing a combined effect of the two drugs *in vivo*. We did, however, find promising *in vitro* drug efficacy with potential for additive inhibitory effects by combining lapaquistat and leading anti-cryptosporidial compounds currently in the development pipeline (Figures S5J–S5L).

GSH is the primary metabolite in the defense against oxidative damage in cells across all kingdoms of life.^38–40^ Remarkably, we found that while *Cryptosporidium* carries genes to utilize GSH, in contrast to most eukaryotes, it does not have the genes for GSH synthesis. In fact, as of this writing, *Cryptosporidium* appears to be the only intracellular parasite that relies on host GSH reserves. When host GSH synthesis is blocked via chemical inhibitors such as erastin or BSO, *Cryptosporidium* growth and development are restricted. *Cryptosporidium* has an extremely streamlined genome and carries almost 200 putative transporters to obtain various amino acids, nucleotides, sugars, and other metabolites that it cannot synthesize from its host cell.^41–43^ While no definitive GSH transporters have been identified, orthologous *Cryptosporidium* gene candidates in the major facilitator superfamily (MFS) of transporters (cgd3_3100 and cgd8_3910) with at least 23% sequence identity to a recently discovered GSH transporter in bacteria^44^ warrant further study. It has been shown that loss of GSH synthesis genes is detrimental to the asexual stages of related parasites *Plasmodium*^45^ and *Toxoplasma*^46^; however, their importance for sexual stage development is unknown. *Cryptosporidium* expresses its *GR* gene to recycle GSH throughout its asexual and sexual life cycle, yet two GSTs, enzymes that post-translationally modify proteins with GSH, appear to be expressed exclusively in female (cgd7_4780) and male (cgd8_2970) parasites.^18^ The redox state, particularly GSH levels, of mouse^47,48^ and human oocytes^49^ and sperm^50^ has been shown to be important for their viability and motility, respectively, with the ability to affect the rate of successful fertilization and blastocyst formation. Our observation of greater male egress under a reducing environment aligns with this, suggesting that maintenance of proper redox balance in germ cells may be a more widespread requirement in eukaryotes.

A vast and robust dataset on *Cryptosporidium*-host dependencies and interactions generated by this whole genome KO study awaits further inquiry. Our dataset includes other parasite infection phenotypes such as the recruitment of host actin beneath the parasite, parasite vacuole size, and host viability data, revealing many other host genes that affect these aspects of *Cryptosporidium* infection when knocked out, lighting the way for follow-up studies. Interestingly, we found that host genes involved in the maintenance of epithelial cell-cell junctions, such as *ZO-1, JAM-A*, and *E-cadherin*, were also important for *Cryptosporidium* growth and female development, producing a significant increase in smaller, less developed parasites when disrupted, while also having relatively minimal effects on host cell viability. On the pathogen side, these results also allow host factors affecting sexual stage development to now be dissected away from the contexts of parasite entry and actin pedestal formation, since a negative effect on one infection phenotype did not necessarily always result in a negative effect on another. For example, knocking out components of the proton-pumping vacuolar-type ATPases (V-ATPases) enhanced parasite numbers; however, progression to female development was slightly reduced. V-ATPases are membrane-bound proton pumps with essential functions in basic physiology and infection, particularly in the functioning of the lysosome.^51^ Lysosomes are major players in innate cellular defense against invading pathogens, representing a promising avenue of future *Cryptosporidium*-host interaction research.

Our arrayed CRISPR screen allowed us to not only discover a requirement for host GSH by *Cryptosporidium* but also uncover the significant antioxidant properties of squalene in intestinal epithelial cells. In mice and humans, *Cryptosporidium* predominantly infects the ileum,^52,53^ the most distal section of the small intestine, although a reason for this preference has been elusive. A recent transcriptomics analysis of the mouse and human small intestine found the ileum to be the site of maximal expression of cholesterol biosynthesis genes in both species.^54^ Higher expression of cholesterol biosynthesis genes implies a higher flux through its intermediates, such as squalene, in the ileum. Our observations that *Cryptosporidium* survival in the intestine is influenced by these cholesterol intermediate metabolites may help to explain this ileal preference. Finally, from the host perspective, it will be intriguing to explore how the antioxidant effects of a squalene accumulation in the intestine may be used to our advantage. ROS, suppression of antioxidant pathways, or a lack of reduced GSH have been identified as instigators or components of many pathologies, including inflammatory bowel disease (IBD),^55^ environmental enteropathy,^56,57^ and cancer.^58,59^ Modulating the redox state of the intestine by administering compounds capable of increasing squalene levels may prove to be effective against the development or progression of these pathologies. The *Cryptosporidium* parasite has a broad host range yet has evolved to infect a very specific cell type, intestinal epithelial cells, manipulating and remodeling them to support its growth and development. As we have demonstrated here, studying the interactions of this parasite with its host can be an effective and powerful tool to better understand small intestinal and epithelial cell biology.

### Limitations of the study

There are a few limitations inherent to the highly technical screening approach we have used. While we rigorously measured the efficacy of transfection and knockdown of our controls, we cannot know the efficacy of all the targeted crRNA guides we have used (in combination). Therefore, our screen is prone to false negatives—genes that do not show an infection phenotype due to inefficient KO of the target host gene. The host cells we have used for our screen are HCT-8s, an immortalized intestinal epithelial cell line, grown under standard cell culture conditions. Within this cell culture model, we could not recapitulate the complexity of the intestine, with its different cell types, and the exact nutrient or oxygen saturation conditions within the small intestine. One final limitation that is worth considering is the strain of *Cryptosporidium* parasites used in the study. For this screen, we used the *Cryptosporidium parvum* IOWA IIa strain. The natural host of this species is bovine, but it can also infect humans. *Cryptosporidium hominis* is the species that naturally infects humans; however, it would have been almost impossible to obtain *C. hominis* in high enough amounts required to perform a screen of this scale, and it is closely related (95%–98% genetically identical) to the species used in this study.

## Resource Availability

### Lead contact

Further information and requests for parasite strains or cell lines can be directed to the lead contact, Adam Sateriale (adam.sateriale@crick.ac.uk).

### Materials availability

Parasite lines, vectors, and cell lines generated for this study are available on request.

## Star★Methods

### Key Resources Table

**Table:** 

REAGENT or RESOURCE	SOURCE	IDENTIFIER
Antibodies		
Anti-COWP1	Kind gift of Boris Striepen	N/A
Anti-DMC1	Kind gift of Chris Huston	N/A
Anti-Lamin A/C	Santa Cruz Biotechnology	Cat# sc7292; RRID: AB_627875
Anti-Ezrin	Cell Signaling Technology	Cat# 3145; RRID: AB_2100309
Anti-HA	Roche	Cat# 11867423001; RRID: AB_390918
Anti-SQLE	Proteintech	Cat# 12544-1-AP; RRID: AB_3672543
Anti-Cas9	Sigma-Aldrich	Cat# SAB4200701; RRID: AB_2891217
Anti-alpha tubulin	Abcam	Cat# ab7291; RRID: AB_2241126
Bacterial and virus strains		
NEB 5-alpha competent *E. coli*	NEB	Cat# C2987H
Chemicals, peptides, and recombinant proteins		
*Vicia villosa* lectin (VVL), fluorescein conjugate	Vector Laboratories	Cat# FL-1231-2
*Helix pomatia* lectin (HPA), Alexa Fluor™ 647 conjugate	Invitrogen	Cat# L32454
Hoechst 33342	Invitrogen	Cat# H3570
Phalloidin, Alexa Fluor™ 546 conjugate	Invitrogen	Cat# A22283
Phalloidin, Alexa Fluor™ 647 conjugate	Invitrogen	Cat# A22287
BODIPY™-505/515	Invitrogen	Cat# D3921
Doxycycline hydrochloride	Sigma-Aldrich	Cat# D3072
Paromomycin sulfate	Biosynth	Cat# AP31110
Rapamycin	Apex Bio	Cat# A8167
Atorvastatin	Selleck Chemicals	Cat#S5715
Lovastatin	Merck	Cat# PHR1285
Lapaquistat acetate	MedChemExpress	Cat# HY-16275
Lapaquistat acetate	Enamine	N/A (Custom order)
NB-598	Apex Bio	Cat# A3646
Ro 48-8071	MedChemExpress	Cat# HY-18630A
N-acetyl cysteine	Sigma-Aldrich	Cat# A9165
Glutathione ethyl ester	Cambridge Bioscience	Cat# CAY14953
Erastin	Selleck Chemicals	Cat# S7242
Menadione	Sigma-Aldrich	Cat# M5625
IPP	Sigma-Aldrich	Cat# I0503
GGPP	Sigma-Aldrich	Cat# G6025
Methyl cellulose	Sigma-Aldrich	Cat# M0512
AD4	Sigma-Aldrich	Cat# A0737
Tocopherol	Sigma-Aldrich	Cat# 258024
Ascorbic acid	Sigma-Aldrich	Cat# A4544
BSO	Tocris Bioscience	Cat# 6954
Auranofin	Selleck Chemicals	Cat# S4307
FINO2	MedChemExpress	Cat# HY-129457
RSL3	Apex Bio	Cat# B6095
Critical commercial assays		
CellROX™ Green	Invitrogen	Cat# C10444
CellROX™ Deep Red	Invitrogen	Cat# C10422
GSH/GSSG-Glo™ assay	Promega	Cat# V6611
NanoGlo Luciferase kit	Promega	Cat# N1150
RNeasy Mini kit	Qiagen	Cat# 74104
Watchmaker mRNA Library Prep kit	Watchmaker Genomics	Cat# 7BK0001
Deposited data		
Image analysis data from arrayed CRISPR screen	This study; Table S2	N/A
Experimental models: Cell lines		
HCT-8	ATCC	CCL-244
HCT-8 Cas9	This study	N/A
HCT-8 SQLE-KO	This study	N/A
Experimental models: Organisms/strains		
IFNγ KO mice	The Jackson Laboratory	Strain# :002287; RRID: IMSR_JAX:002287
IFNγR KO mice	The Jackson Laboratory	Strain #:003288; RRID: IMSR_JAX:003288
*Cryptosporidium parvum*	Bunch Grass Farms, ID, USA	IOWA strain IIa
Oligonucleotides		
For crRNA sequences used in the CRISPR screen, see Table S2	Dharmacon	N/A
For primers and guide RNAs used for recombinant *C. parvum* creation, see Table S3	This study	N/A
Edit-R tracrRNA	Dharmacon	Cat# CM-009646-01
SQLE crRNA 1	Dharmacon	Cat# CM-009646-02
SQLE crRNA 2	Dharmacon	Cat# CM-009646-02
SQLE crRNA 3	Dharmacon	Cat# CM-009646-03
SQLE crRNA 4	Dharmacon	Cat# CM-009646-04
SQLE crRNA 5	Dharmacon	Cat# CM-009646-05
Recombinant DNA		
Genome-CRISP™ Inducible Cas9 human AAVS1 Safe Harbor knock-in kit	GeneCopoeia	Cat# SH016
CpLIC mCherry with Nanoluciferase and Neomycin^R^ plasmid	Kind gift of Boris Striepen	N/A
CpLIC HA-mNeonGreen with Nanoluciferase and Neomycin^R^ plasmid	This study	N/A
CpLIC DiCre with Nanoluciferase and Neomycin^R^ plasmid	This study	N/A
TK repair plasmid with mCherry, Nanoluciferase, and Neomycin^R^	This study	N/A
COWP1-HA plasmid with Nanoluciferase and Neomycin^R^	This study	N/A
HAP2-HA repair plasmid with Nanoluciferase and Neomycin^R^	This study	N/A
GlutR repair plasmid with DiCre, Nanoluciferase, and Neomycin^R^	This study	N/A
Software and algorithms		
Harmony v5.0	Revvity	https://www.revvity.com/gb-en/product/harmony-5-2-office-revvity-hh17000019
ImageJ v2.1.0/1.53c	ImageJ	https://imagej.net/
PRISM V10.2.3	GraphPad	https://www.graphpad.com/features
R v.4.4.1	R Core Team	https://www.r-project.org/
FastQC V0.11.7	Babraham Bioinformatics	https://www.bioinformatics.babraham.ac.uk/projects/fastqc/
kallisto V0.45.0	Bray et al.^60^	https://pachterlab.github.io/kallisto/
MassHunter vB.07.02.1938	Agilent	https://www.agilent.com/en/promotions/masshunter-mass-spec
MANIC V3.0.21	Adapted from Behrends et al.^61^	N/A
Other		
RPMI-1640 medium	Gibco	Cat# A10491
Penicillin-Streptomycin	Gibco	Cat# 15140122
Amphotericin B	Sigma-Aldrich	Cat# A2942
ProLong™ Gold Antifade Mountant	Invitrogen	Cat# P36934
DharmaFECT 2	Dharmacon	Cat# T-2002
PrimeSTAR Max DNA Polymerase	Takara	Cat# R045B

### Experimental Model and Study Participant Details

#### Human cell lines

HCT-8 cells were obtained from the ATCC, authenticated by STR profiling, and maintained by Cell Sciences at The Francis Crick Institute. HCT-8 Cas9 and HCT-8 SQLE-KO cell lines were generated for this study. Cells were grown at 37°C in an atmosphere with 5% CO_2_ in RPMI 1640 media supplemented with 10% fetal bovine serum (FBS), penicillin/streptomycin, and amphotericin B. During *Cryptosporidium* infections, media was changed to RPMI with 1% FBS. Cell lines were routinely tested for the presence of mycoplasma.

#### Mouse strains

*IFN*γ^-/-^ or *IFN*γ*R*^-/-^ mice were bred and maintained by the Biological Research Facility at the Francis Crick Institute. Mice were housed in individually vented cages under a 12 h light/dark cycle with access to food and water *ad libitum*. 4–8 week-old littermates of either sex were randomly assigned to experimental groups. Experiments were undertaken in accordance with UK Home Office regulations under a project license to A.S. (PP8575470).

#### Parasite strains

*Cryptosporidium parvum* oocysts (IOWA strain IIa) were purchased from Bunchgrass Farms, ID, USA. COWP1-HA-mNeonGreen, HAP2-HA, inducible GR-KO, and the mouse-adapted *C. parvum*-mCherry transgenic parasites were created and passaged using 4-8 week-old *IFN*γ^-/-^ mice.

### Method Details

#### Generation of transgenic *Cryptosporidium* parasites

All transgenic parasites were created with IOWA strain IIa (Bunch Grass Farms, ID, USA) as the parental line using methods described previously.^62,63^ Briefly, 2.5 x 10^7^ oocysts were induced to excyst following 1% sodium hypochlorite treatment and incubation in 0.75% sodium taurocholate in RPMI for 45 minutes at 37°C to release sporozoites. Sporozoites were transfected with a plasmid expressing Cas9 and a guide RNA targeting the desired region, along with a linear DNA template for repair of the Cas9-induced double-strand break by homology-directed repair. This linear fragment contained the tag of choice (3x HA for COWP1-HA and HAP2-HA parasite lines) or loxP-flanked recodonised genetic regions and split Cre recombinase (DiCre) genes (inducible GR-KO line), as well as genes for neomycin resistance and nanoluciferase expression, flanked by 50 bp regions of homology to the cut site. NEB 5-alpha competent *E. coli* were used to propagate Cas9 and repair template plasmids. Transfected sporozoites were used to infect *IFN*γ^–/–^ mice by oral gavage as described previously.^63^ Mice were then given paromomycin in their drinking water (16 mg/mL) to select for transgenic parasites. For details of guide RNAs and primers used for cloning the repair templates, please see Table S3.

#### Generation of inducible Cas9-expressing HCT-8 cells

A tetracycline-inducible Cas9-expressing HCT-8 cell line was generated using the Genome-Crisp™ human AAVS1 Safe Harbour knock-in kit with puromycin selection (kit no. SH016; SH100 for AAVS1 CRISPR-Cas9 clone and SH304 for AAVS1 Cas9 knock-in donor clone-TRE3G-Puro from GeneCopoeia). Briefly, sgRNA targeting the AAVS1 site on human chromosome 19 was used to initiate a Cas9-mediated double-strand break, allowing for insertion of a single copy of a tet-inducible Cas9-coding repair cassette. Positive clones were selected for by puromycin treatment, single-cell sorting, cell expansion, and confirmation by gene amplification and sequencing for correct gene cassette insertion, and immunoblotting for expression of the Cas9 protein after doxycycline treatment. Growth rates and susceptibilities to *Cryptosporidium* infection of selected positive clones were compared to the parental HCT-8 cell line to ensure there were no drastic changes in infection phenotypes (Figures S1A–S1C). A single clone (G09) was used for all screening experiments.

#### Arrayed genome-wide CRISPR-Cas9 screen

We first assessed the levels of protein knockdown that could be achieved in a 384-well plate format by using test crRNA guides against a few genes with reliable protein-directed antibodies. When we seeded Cas9-expressing HCT-8 cells (induced using 0.5 μg/mL doxycycline) along with transfection reagent Dharmafect 2 (0.045 μL in 2.5 μL Opti-MEM per well) into wells containing 200 nM tracrRNA and 200 nM pooled crRNA guides against a particular gene, we found protein levels reduced by 70-80% three days later, as indicated by staining with anti-lamin or anti-ezrin antibodies (Figure S1D).

Cells could then be infected by *Cryptosporidium* oocysts following standard infection procedures as described previously.^62^ Briefly, oocysts were treated with a 1% sodium hypochlorite solution for 5 minutes at 4°C, followed by washing once in PBS. Oocyst excystation was induced by incubating in 0.75% sodium taurocholate in RPMI for 10 minutes at 37°C. Oocysts were resuspended in 1% RPMI before addition to wells. During initial troubleshooting for setting up the screen, we analysed COWP1-expression over a 45-55-hour time window and discovered that the highest percentage of COWP1-positive parasites were present 49 hours post-infection. Moving forward, we fixed infected cells at 49 h post-infection in 4% formaldehyde. After fixing for 15-20 minutes, cells were washed in PBS, permeabilised in 0.25% Triton X-100, and blocked in 4% BSA. For convenient immunofluorescence-based analysis, we populated the four available imaging channels by staining for parasite vacuoles (using the Fluorescein-conjugated lectin *Vicia villosa* lectin or VVL), female parasites (detected with an antibody against female-specific oocyst wall protein COWP1), host-derived actin ‘pedestals’ formed beneath parasite vacuoles (detected by fluorescently-conjugated phalloidin), and host cell nuclei (using Hoechst) (Figure S1E). Specifically, we stained female parasites first using rat anti-COWP1 (1:700; created for this study) antibodies with an overnight stain at 4°C, followed by counter-staining with goat anti-rat AlexaFluor-647 (1:1000; Invitrogen), VVL-fluorescein (1:5000; VectorLabs), and Hoechst (1:10,000; ThermoFisher). Fixed and stained plates were imaged using an Opera Phenix High Content Screening system (Revvity). Wells were imaged using a 20 x NA 0.8 air lens. Five fields of view per well were acquired. To obtain both host cell and apically-located intracellular parasite information, three z-stacks with a step size of 3 μm were taken using 405, 488, 568, and 640 nm excitation lasers paired with 450, 540, 600, and 690 nm emission filters. Image analyses were done using Harmony software v5.0 (Revvity) and detailed steps are further described in Table S1.

We defined the ideal number of parasites needed to achieve a rate of infection that was reliably detectable by our high-content imaging platform, with room for the infection rate to significantly decrease or increase and still be detectable, while also not causing too much host cell death. To this end, we conducted titrations of each oocyst batch commercially obtained (as their viabilities often vary from batch to batch) to define these parameters. As we increased the number of oocysts, there was a corresponding decrease in host cell viability (Figure S1F). As a result, we aimed for an intermediate multiplicity of infection (MOI) of 0.5 (or 50% of host cells infected) 49 hpi to allow room for a reliable detection of host factors that may increase or decrease parasite numbers without compromising host cell viability. Normally, the percentage of female parasites in the population at 49 h post-infection remained constant, while the percentage of parasite vacuoles with detected actin pedestals slightly increased, as the MOI of infection increased (Figure S1G). For the full genome screen, we aimed to conduct an arrayed knockout of more than 18,000 host genes – representing 92 % of annotated protein-coding human genes – in triplicate. This amounted to more than 180 384-well plates for the full screen. To minimise variability and maximise confidence in each plate, we decided to include positive and negative control guide RNAs in each plate. However, as very little was known about host genes that influence *Cryptosporidium* infection, we first conducted a pilot screen with a smaller crRNA library against select genes, in the hopes of discovering appropriate controls. The pilot screen revealed some host genes that increased or decreased *Cryptosporidium* growth when knocked out (Figure S1H), from which we selected *RhoA* and *Rab8a* as our controls, as disabling these genes decreased and increased parasite growth respectively.

For the full genome screen, only protein-coding genes were targeted, and crRNA guides were designed against the GRCh38.p14 reference human genome. Guides were bought from Dharmacon (now Horizon Discovery) as part of multiple libraries covering 18,252 genes and assembled into a 384-well format in-house along with positive and negative controls per plate (described in Figure S1I). Four guides per gene were pooled (200 nM in total), or positive and negative control crRNAs (200 nM each) were deposited per well, along with 200 nM tracrRNA in each 384-well plate. HCT-8 cells induced to express Cas9 were then seeded into these wells, along with transfection reagent Dharmafect 2 (0.045 μL in 2.5 μL Opti-MEM per well), and allowed to grow for three days. Cells were then infected with *C. parvum* (to achieve a parasite/host nuclei percentage, or MOI, of 50% after 49 hours, followed by fixation, immunofluorescence labelling, and high-content imaging as described above. Triplicates of each plate were created, allowing us to calculate median z-scores for each infection parameter (outlined in Figure S1E), for each host gene. Plotting all the z-score values for the full screen for the parasite growth parameter (parasites/host nuclei), we found that our positive and negative controls cluster at either ends of the growth spectrum, our non-targeting guides clustered around non-significant z-scores (-2 to 2 exclusive), with our on-targeting guides normally distributed along this entire spectrum (Figure S1J). All crRNA guide sequences, raw data for infection phenotypes, and z-scores calculated for each replicate for each gene KO are provided in Table S2. Subsequent to performing the screen, we conducted an additional arrayed CRISPR screen of 214 genes absent from the original library, along with non-targeting crRNA controls under the same transfection and infection conditions. However this produced no significant findings and did not affect our original results in any way. Raw data obtained from this screen is provided in Table S4.

Knocking out many host genes expectedly reduced the viability of HCT-8 cells (Figure S1L). However, as we did not conduct a parallel screen without infection, we are unable to say whether the loss of host viability was only due to the importance of the gene for the viability of the cell, or whether the compounded effect of infection and host gene knockout caused cell death. As a result, we applied a z-score cut-off for cell viability before conducting downstream STRING-based analyses to avoid skewing the assessment of our other infection parameters. We compared these genes, assumed to be essential based on how they affected host cell viability in our screen, to publicly available datasets^64,65^ that have conducted meta-analyses of multiple CRISPR screens to infer gene essentiality across several different cell types. We found almost 80% of genes that we excluded were considered essential in at least one of these datasets (Table S5). We functionally characterised these genes using the STRING database (v 12.0), and discovered a majority belonged to critical cellular components such as the ribosome, proteasome, and cell cycle regulators (Figures S1M and S1N).

#### CRISPR screen data analysis

Output from Harmony was collated into a single table and any features relating to total counts were corrected if they had fewer than five imaged fields by taking the average value per field and multiplying by five. Edge-effects were then corrected through positional normalisation per feature by taking the outer product of the row and column medians across all the plates to create an effect-matrix, scaling this by the feature mean and then multiplying the screen as a [plate, row, column] array by the scaled effect-matrix. Robust z-scores were calculated for features using the median and absolute-median-deviation of the sample wells per plate.

#### Generation of SQLE-KO HCT-8 cells

Five different crRNA guides (Horizon Discovery) against human *SQLE* (listed in the Key Resources Table) were pooled to a final concentration of 200 nM and used to transfect Cas9-expressing HCT-8 cells along with 200 nM tracrRNA. 3 days post-transfection, cells were sorted by FACS to grow single clones, which were assessed on a build-up of neutral lipids by BODIPY-505 staining, followed by immunoblotting for expression of SQLE and sequencing of the *SQLE* gene locus to confirm Cas9-assisted partial gene deletion. Three positive clonal populations were identified and verified this way, and all downstream experiments were carried out with clone F02.

#### Immunofluorescence-based parasite growth assays

All drug-treatment assays were conducted in 96- or 384-well plates. HCT-8 cells were seeded 48 h prior to infection. Oocysts were treated as described above prior to host cell infection. For drug treatments, compounds were added 2-3 hours post-infection, except for invasion assays, in which case cells were pre-treated with the drug 16-24 h before infection. For drug synergy testing, compounds were added immediately prior to infection. Cells were fixed 6, 24, or 49 h post-infection in 4% formaldehyde, washed in PBS, permeabilised in 0.25% Triton X-100, and blocked in 4% BSA. Female parasites were detected using mouse anti-DMC1 (1:1000) for 1 hour, followed by counter-staining with goat anti-mouse AlexaFluor-647 (1:1000), VVL-Fluorescein (1:5000), or *Helix pomatia* agglutinin (HPA)-AlexaFluor™ 647 (1:5000) to detect all parasites, and Hoechst (1:10,000) to detect host nuclei. HPA is a lectin that, like VVL, detects α-*N*-acetylgalactosamine residues and so we use VVL and HPA interchangeably to detect parasites. Antibodies were obtained from Invitrogen unless otherwise stated. Plates were read using a BioTek Cytation 5 (Agilent Technologies) wide-field imaging reader with a 20x air objective. Four fields of view per culture well were imaged, and parasites and host nuclei were counted using its Gen5 software.

For higher-resolution imaging to stage asexual parasites and count male parasites, HCT-8 cells were seeded onto glass coverslips in 24-well plates and infected and drug-treated as previously described. Following fixation and staining as above, coverslips were mounted on slides with ProLong Gold antifade (Invitrogen) and imaged using a VisiTech iSIM for super-resolution imaging (100x or 150x oil immersion objectives, Olympus). Z-stacks of 0.2 μm step-size spanning a width of 5 μm were taken to capture the most number of parasites per field of view, and maximum intensity projections were created in ImageJ (version 2.1.0/1.53c).

To count males, infected cells were fixed at 45 hpi to ensure a mixed population of all stages were present. To quantify the percentage of all males in the population, early males (8n), late-stage males (16n) and ‘empty’ male vacuoles were counted as individual parasites, along with females and asexual stages. At least 200 parasites were counted per replicate per condition. To quantify the percentage of egressed males in the population, individual egressed males were each counted as one parasite.

#### RNA isolation and bioinformatic analyses

WT and SQLE-KO HCT-8 cells were infected following standard infection procedures as described previously. 1 x 10^6^ *C. parvum* oocysts were used to infect wells of a 6-well plate seeded with 6 x 10^5^ parental or SQLE-KO cells 48 h prior. 49 hours post-infection, RNA was recovered using the RNeasy Mini Kit (Qiagen). Total RNA was quantified by Bioanalyzer and Qubit (Agilent, Thermo). Ribosomal RNA was depleted and libraries were constructed using the Watchmaker mRNA kit (Watchmaker Genomics) according to the manufacturer’s instructions. The libraries were then pooled and sequenced on an Illumina NovaSeq X using 100bp paired-end read chemistry.

Raw sequencing reads were first assessed using FastQC (version 0.11.7) (https://www.bioinformatics.babraham.ac.uk/projects/fastqc/). Sequencing reads from each RNAseq sample were pseudo-aligned to both the predicted *C. parvum* full-length transcripts (CryptoDB-64_CparvumIOWA-ATCC_AnnotatedTranscripts) downloaded from CryptoDB^66^ (Release 64, accessed Aug 2023) and human genome transcripts (GRCh38.p14 Transcript sequences) downloaded from GENCODE^67^ (Human Release 46, accessed Sept 2024) using *kallisto*^60^ (v. 0.45.0). Briefly, the references were first prepared using *kallisto index*, after which paired end data were pseudo-aligned to the indexed reference using *kallisto quant* with default parameters.

RNA-seq data were analysed in *R* (v.4.4.1); data exploration, filtering, and differential gene expression analyses were performed using DESeq2^68^ (v.1.46.0). Raw counts were filtered to remove low-abundance transcripts and differentially expressed transcripts were determined using a Wald test of raw counts between infected cell conditions. Transcripts were defined as being significantly differentially expressed based on fold change (log_2_(FC) > 1 or < -1) using *apeglm*^69^ and a Benjamini-Hochberg adjusted p value (*P*adj < 0.05). Volcano plots were used to visualise differentially expressed transcripts.

#### Cellular ROS measurements

Host cells were either infected and drug-treated for 48 hours (HCT-8 only) or uninfected (parental HCT-8 vs SQLE-KO). CellROX™ Green or CellROX™ DeepRed dyes were added to wells at a final concentration of 5 μM and incubated for 30 minutes at 37°C. Cells were then washed three times in PBS, fixed, stained with nuclear dye Hoechst and imaged using a BioTek Cytation 5 (Agilent Technologies) to obtain total fluorescence intensities and area covered to obtain mean fluorescence intensities (MFI).

#### GSH-GSSG ratio measurements

The ratio of reduced to oxidised glutathione (GSH) in Cas9-HCT8 (clone G09) and SQLE-KO cells (clone F02) was quantified using the GSH/GSSG-Glo™ Assay kit (Promega) based on provided instructions. Briefly, cells were treated with indicated drugs in 1% FCS RPMI. 4 hours post-treatment, media was removed, and cells were lysed in either Total or Oxidised Glutathione reagents, shaken and then treated with Luciferin Generation reagent. 30 minutes later, Luciferin Detection reagent was added to all wells and luciferase readings were taken using a BioTek Cytation 5 (Agilent Technologies) plate reader.

#### Quantification of cholesterol and squalene in HCT-8 cells and oocysts

Quantification of squalene and cholesterol in host cells or *Cryptosporidium* oocysts was performed by gas chromatography-mass spectrometry (GC-MS). For drug treatments of HCT-8 cells, 300,000 cells of either WT or SQLE-KO HCT-8 cells were seeded in 6-well plates. Two days later, 1% FCS RPMI media containing the drug or vehicle were added. 48 hours later, cells were processed for extraction of apolar metabolites as follows.

Briefly, media was removed and plates placed on ice and washed twice with ice-cold PBS. Cells were scraped into 250 μL chloroform:methanol:water (1:3:1, v/v) containing an internal standard (5 nmol ergosterol and 1 nmol tetracosane for sterol and squalene analysis, respectively), and incubated in a waterbath at 4°C for 1 hr with 3 x 8 minutes pulse sonication. *Cryptosporidium* oocysts were washed in ice-cold PBS before addition of the solvents and sonication as above. Samples were spun at 13,200 rpm for 10 minutes at 4°C and supernatant (metabolite extract) was transferred to new tubes and dried in a rotary vacuum concentrator. 250 μL chloroform:methanol:water (1:3:1, v/v) was added to dried samples, followed by 100 μL water (for sterols only: containing a second standard of 5 nmol lanosterol). Samples were briefly vortexed and centrifuged (13,200 rpm, 10 minutes, 4°C) to partition polar and apolar metabolites. The lower apolar phase (containing sterols and squalene) was transferred to a glass vial insert and dried once more, ready for analysis.

Data acquisition was performed using an Agilent 7890B-7000C GC-MS system in EI mode after resuspension of twice methanol-washed dried extracts in either (a) for sterols: 25 μL BSTFA + TMCS derivatisation reagent (Sigma, RT, >1 hr) or (b) for squalene, 25 μL hexane (Fisher, RT). GC-MS parameters were as follows: for sterols: carrier gas, helium; flow rate, 0.9 mL/min; column, DB-5MS+DG (30 m + 10 m × 0.25 mm, Agilent); inlet, 270°C; injection volume, 1 μl; temperature gradient, 80°C (2 min), ramp to 140°C (30°C/min), ramp to 250°C (5°C/min), ramp to 320°C (15°C/min, 6 min hold); scan range was m/z 40–600. Data were acquired using MassHunter software (version B.07.02.1938). For squalene, parameters were as above, except: inlet, 250°C; temperature gradient, 70°C (1 min), ramp to 230°C (15°C/min, 2 min hold), ramp to 325°C (25°C/min, 3 min hold); scan range was m/z 40-565.

Data analysis was performed using MANIC software (v3.0.21), an in house-developed adaptation of the GAVIN package.^61^ Metabolites were identified and quantified by comparison to authentic standards. All data are presented as mean ± standard deviation (SD) of 6 replicates for each condition. P-values calculated by ordinary one-way ANOVA.

#### Mouse drug treatments

4-6 week-old *IFN*γ*R*^-/-^ mice were infected with equal amounts of a mouse-adapted strain of *C. parvum* (expressing mCherry and nanoluciferase).^70^ Atorvastatin or lapaquistat (were administered to mice once daily by oral gavage in a suspension of 0.5% carboxymethylcellulose as the vehicle. Doses of lapaquistat at 200 μg or 600 μg were calculated by allometric scaling from the human doses tested during Phase 2/3 trials of lapaquistat.^28^ For mice infected with the inducible GR-KO parasites, rapamycin (0.05 mg/mL) was administered in their drinking water. Nanoluciferase levels in fecal samples were tracked as a proxy for parasite burden as previously described^52^ using a NanoGlo luciferase kit (Promega).

### Quantification and Statistical Analysis

The number of mice, culture wells per experiment, or experimental repeats are described for each result in their corresponding figure legends, along with the details of statistical tests used for each comparison. Statistical analyses were carried out using GraphPad PRISM (v10.2.3). A Shapiro-Wilk test was used to determine normality of the data presented in figures. Data in Figure 3F did not past this test and a Mann-Whitney test was performed. Numerical p-values for each comparison are provided in the figures; a p-value of less that 0.05 and z-score +/-2 was considered statistically significant for data interpretation in-text. ‘SD’ stands for standard deviation and ‘SEM’ stands for standard error of the mean. No statistical tests were done to pre-determine sample size.

## Supplementary Material

Supplemental information can be found online at https://doi.org/10.1016/j.cell.2025.07.001.

Table S1

Table S2

Table S3

Table S4

Table S5

Supplemental figures

## Data Availability

Data generated from image analysis have been provided in Table S2. Raw image files and transcriptomic data can be made available upon request. No new code was created for this study.

## Highlights

- Arrayed CRISPR screens enable image-based analyses of multiple infection phenotypes
- Survival of *Cryptosporidium* hinges on host squalene, a cholesterol precursor
- Squalene regulates the redox status of glutathione, required by *Cryptosporidium*
- Squalene synthase inhibitor lapaquistat restricts *Cryptosporidium* infection in mice

## In brief

An arrayed microscopy-based CRISPR screen revealed host genes affecting multiple infection phenotypes of the intracellular parasite *Cryptosporidium*. Hits in the host cholesterol biosynthesis pathway affecting parasite growth and sexual development exposed a vulnerability that can be leveraged for treatment.
