# Supplementary material for: The essential host genome for Cryptosporidium survival exposes metabolic dependencies that can be leveraged for treatment
Source: Cell. Author manuscript; Available in PMC 2026 Mar 30. (PMC7618951; doi:10.1016/j.cell.2025.07.001)
Supplement: Supplemental figures [file EMS213015-supplement-Supplemental_figures.pdf]

Supplemental figures

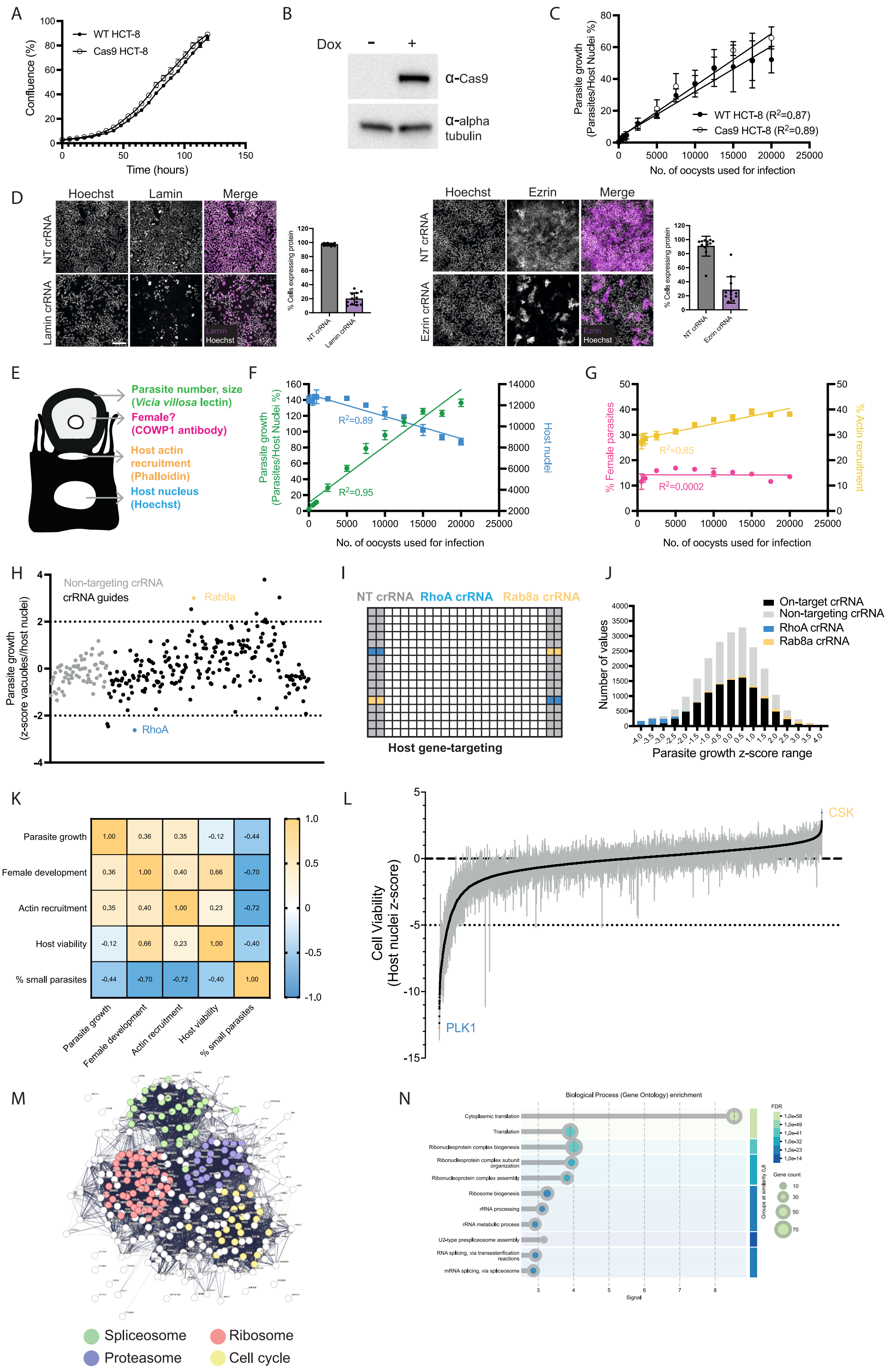

(legend on next page)

Figure S1. Full-genome CRISPR screen setup and validation, related to STAR Methods

(A) Growth comparison of wild-type (WT) and Cas9-HCT-8 cells measured by cell confluency using an Incucyte S3 (Sartorius) over 5 days. Mean and standard error of the mean (SEM) shown for 3 culture wells per condition.

(B) Immunoblot of cell lysates from untreated and doxycycline-treated Cas9-HCT-8 cells to induce Cas9 expression.

(C) Comparison of growth of *C. parvum* in WT and Cas9-HCT-8 cells. Increasing numbers of oocysts were used to infect either cell type. Parasite growth in either cell type (calculated as the number of parasites per host nuclei expressed as a percentage) was assessed 49 hpi. Mean and SD of 5 wells per condition shown.

(D) Levels of protein expression in Cas9-expressing HCT-8 cells 3 days post-transfection of crRNA directed against Lamin (left) or Ezrin (right), measured by immunostaining and calculating fluorescence intensities of each cell over 4 fields of view for 3 wells per condition. Scale bar, 100 μm.

(E) Schematic showing the four parameters of a *C. parvum* infection that were targeted for immunofluorescence analysis in the CRISPR-Cas9 screen: parasite number and size, staining for female parasites, recruitment of host actin to parasite vacuoles, and the number of host nuclei.

(F) Examination of parasite growth (green line) and numbers of host nuclei (blue line) with increasing numbers of oocysts used to infect HCT-8 cells. Note a linear relationship with initial oocyst infection numbers in both cases. Mean and SD of three culture wells per condition. Representative of three independent experiments.

(G) Examination of the percentage of parasite vacuoles with actin pedestals (mustard line) and the percentage of female parasites in the population (magenta line) with increasing numbers of oocysts used to infect HCT-8 cells. Mean and SD of three culture wells per condition. Representative of three independent experiments.

(H) Scatterplot of Z scores from the pilot screen for host genes affecting *C. parvum* growth, highlighting positions of *Rab8a* and *RhoA* above and below the 2/−2 cutoff for significantly affecting growth, resulting in their selection as positive and negative controls for the full-genome CRISPR screen.

(I) Layout for each of the 183 384-well plates used in the full-genome CRISPR screen. Non-targeting control guides (NT crRNA) are placed in gray locations, RhoA controls in blue, Rab8a controls in mustard, and all on-target guides against host genes in white.

(J) Distribution of parasite growth Z scores for the different control and on-target guide RNAs in the full-genome CRISPR screen.

(K) Correlation matrix of different infection parameters analyzed from the screen for selected host genes with a significant Z score in at least one infection parameter.

(L) Rank-ordering of all genes in the screen based on their effect on host cell viability Z scores. Rank-ordering based on median Z scores of three replicates per gene. Variance for each gene shown in gray.

(M) Protein-protein interaction networks of host genes that were excluded from downstream analyses, as they significantly reduced host cell viability when knocked out. Clustering based on KEGG pathway analysis provided by STRING (v 12.0).

(N) Functional clustering of genes that reduced host cell viability by Biological Process Gene Ontology (GO) terms, ranked by their Signal scores provided by STRING. False discovery rates (FDRs) for pathway enrichment are shown for each term.

CellPress
OPEN ACCESS

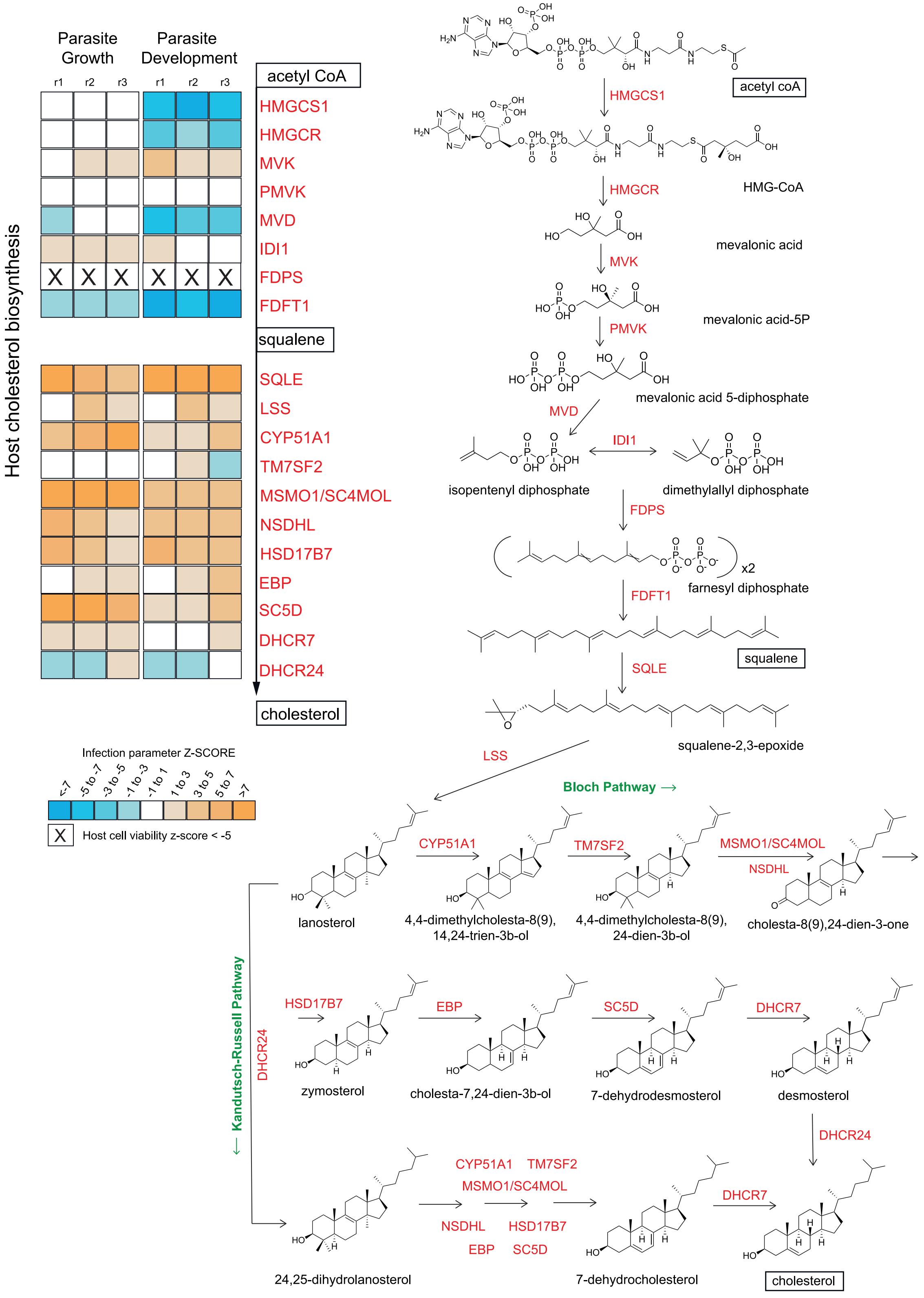

(legend on next page)

Figure S2. Host cholesterol biosynthesis enzymes and their intermediary metabolites, related to Figure 2A

Schematic for the host cholesterol biosynthesis pathway beginning with acetyl-CoA and ending with cholesterol, showing host genes and structures of intermediary metabolites.[22,71] *Z* scores for infection and sexual development parameters for all three replicates for each gene knocked out are depicted on the left. Genes that reduced cell viability by a *Z* score lower than −5 depicted as "X."

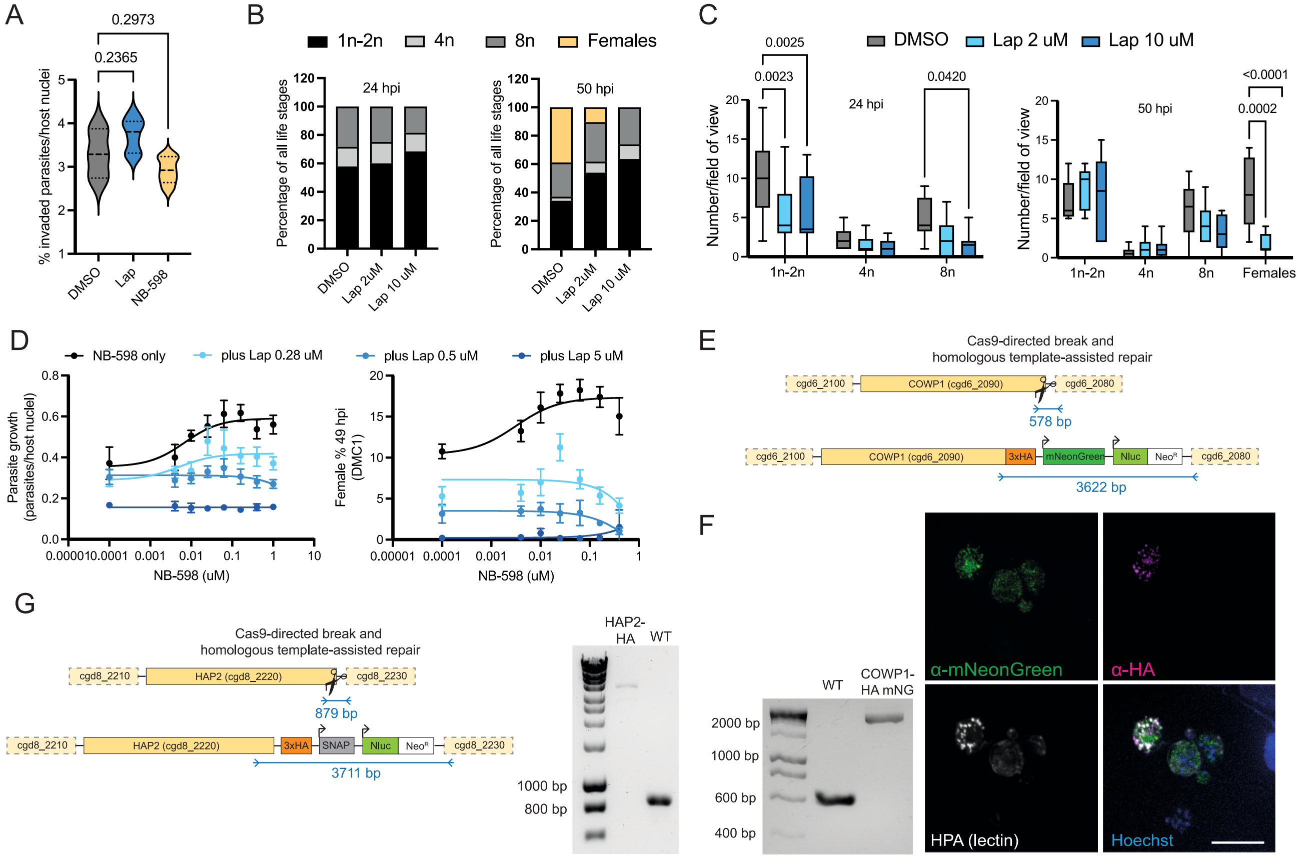

Figure S3. Characterizing modes of action of chemical inhibitors lapaquistat and NB-598, related to Figure 2

(A) Lapaquistat (10 μM) and NB-598 (0.5 μM) do not affect invasion of *C. parvum* sporozoites. HCT-8 cells were treated overnight with the indicated drugs and were infected with newly excysted sporozoites the next day and fixed 4 hpi. Violin plots depict the median value at the heavy dashed line and the lower and upper quartiles at the lighter dashed lines. Data shown represent 6 culture wells per condition. *p* values calculated by one-way ANOVA with Dunnett's multiple comparisons test.

(B) Percentage of life stages seen at 24 hpi in total (left; DMSO = 173 parasites, 2 μM lapaquistat = 140 parasites, 10 μM lapaquistat = 114 parasites) and 50 hpi (right; DMSO = 170 parasites, 2 μM lapaquistat = 115 parasites, 10 μM lapaquistat = 96 parasites) under drug treatment of infected HCT-8 cells.

(C) Number of parasite life stages seen per field of view at 24 and 50 hpi from (B). Mean and SD are shown for at least 8 fields of view per condition. *p* values calculated using two-way ANOVA with Šídák's multiple comparisons test.

(D) Dose-response curves for parasite growth (left) and percentage of females (right) in HCT-8 cells 49 hpi with NB-598 treatment alone (in black) or with increasing concentrations of lapaquistat (in shades of blue). Mean and SD for 3 culture wells per condition shown.

(E) Schematic for the creation of the *C. parvum* transgenic line with endogenously tagged COWP1 by Cas9-assisted break and homology repair.

(F) PCR-based verification of creation of the COWP1-HA-mNeonGreen parasite line (left). Genomic DNA was extracted from either WT parasites or purified transgenic parasites. Immunofluorescence-based verification of the COWP1-HA-mNeonGreen parasite line (right). Transgenic parasites were used to infect HCT-8s, fixed 49 hpi, and probed with an anti-mNeonGreen antibody (green), anti-HA (magenta), *Helix pomatia* agglutinin (HPA)-AlexaFluor™ 647 (white), and Hoechst (blue). Scale bar, 5 μm.

(G) Schematic for the creation of the *C. parvum* transgenic line with endogenously tagged HAP2 by Cas9-assisted break and homology repair (left). PCR-based verification of the creation of the HAP2-HA parasite line (right). Genomic DNA was extracted from either WT parasites or purified transgenic parasites.

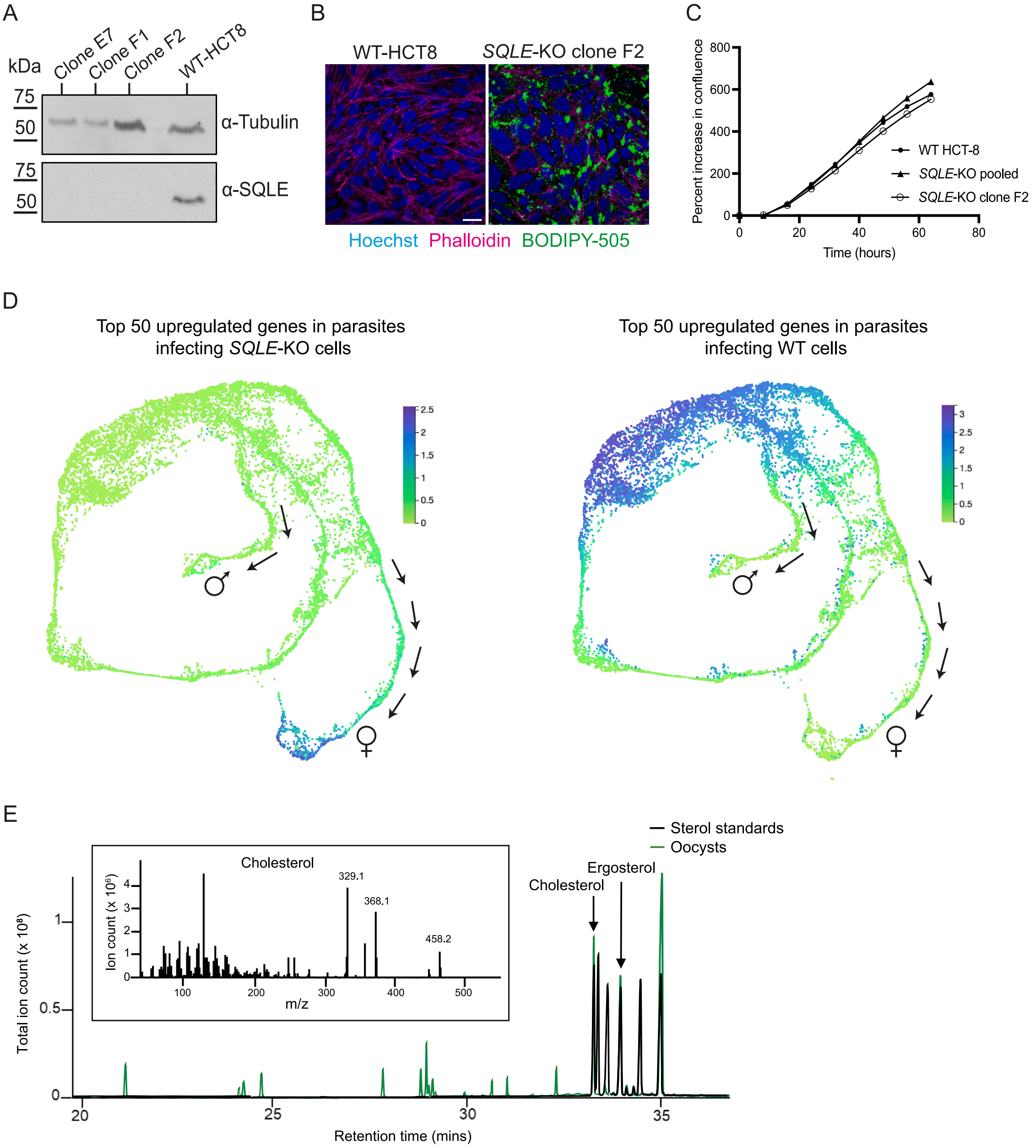

Figure S4. Characterization of SQLE-KO HCT-8 cells and *C. parvum* oocysts, related to Figure 3

(A) Immunoblot for SQLE expression in WT HCT-8 cells and selected SQLE-KO clones.

(B) SQLE-KO cells have a buildup of neutral lipids. Cells were seeded onto coverslips, fixed, and stained to visualize nuclei (Hoechst; blue), actin (Phalloidin-AlexaFluor™ 647; magenta), and neutral lipids (BODIPY-505/515; green). Scale bar, 10 μm.

(C) Growth comparison of WT HCT-8 cells, a mixed population of SQLE-KO cells, and one clonal population of SQLE-KO cells (clone F2) measured by the percent increase of their cell confluency using an Incucyte S3 (Sartorius) over 64 h. Mean shown for 3 culture wells per condition.

(D) Differentially expressed *C. parvum* genes in SQLE-KO and WT HCT-8 infections mapped onto the parasite life cycle. The mean expression for the 50 most significant genes (by adjusted *p* value) with differentially regulated expression was mapped onto the life cycle of *C. parvum*, visually represented as a uniform manifold approximation and projection (UMAP) of single-cell parasite transcriptomes through CZ CELLxGENE.[18,72,73] For parasites infecting SQLE-KO cells, the most significantly upregulated genes cluster to late females (left). Male-specific genes were also significantly upregulated in an SQLE-KO infection (e.g., HAP2 had a $\log_2$FC of 1.6 in *SQLE*-KO vs. WT cells), although to a lesser degree. Late male parasite stages are motile and not anchored to the host cell monolayer; therefore, it is likely many were lost during RNA isolation. For parasites infecting WT cells, significantly upregulated genes compared with SQLE-KO cluster to late-stage asexual meronts (right).

(E) Gas chromatography-mass spectrometry (GC-MS) analysis of *Cryptosporidium* oocysts identifies cholesterol in oocysts. Chromatograms are representative of 6 replicates. The panel shows an overlay of cholesterol analysis chromatograms of *C. parvum* oocysts (green line) and authentic sterol standards (5 nmol, black line). Cholesterol and ergosterol (internal standard) are indicated. A representative spectrum of cholesterol is shown (inset), with diagnostic ions highlighted.

CellPress
OPEN ACCESS

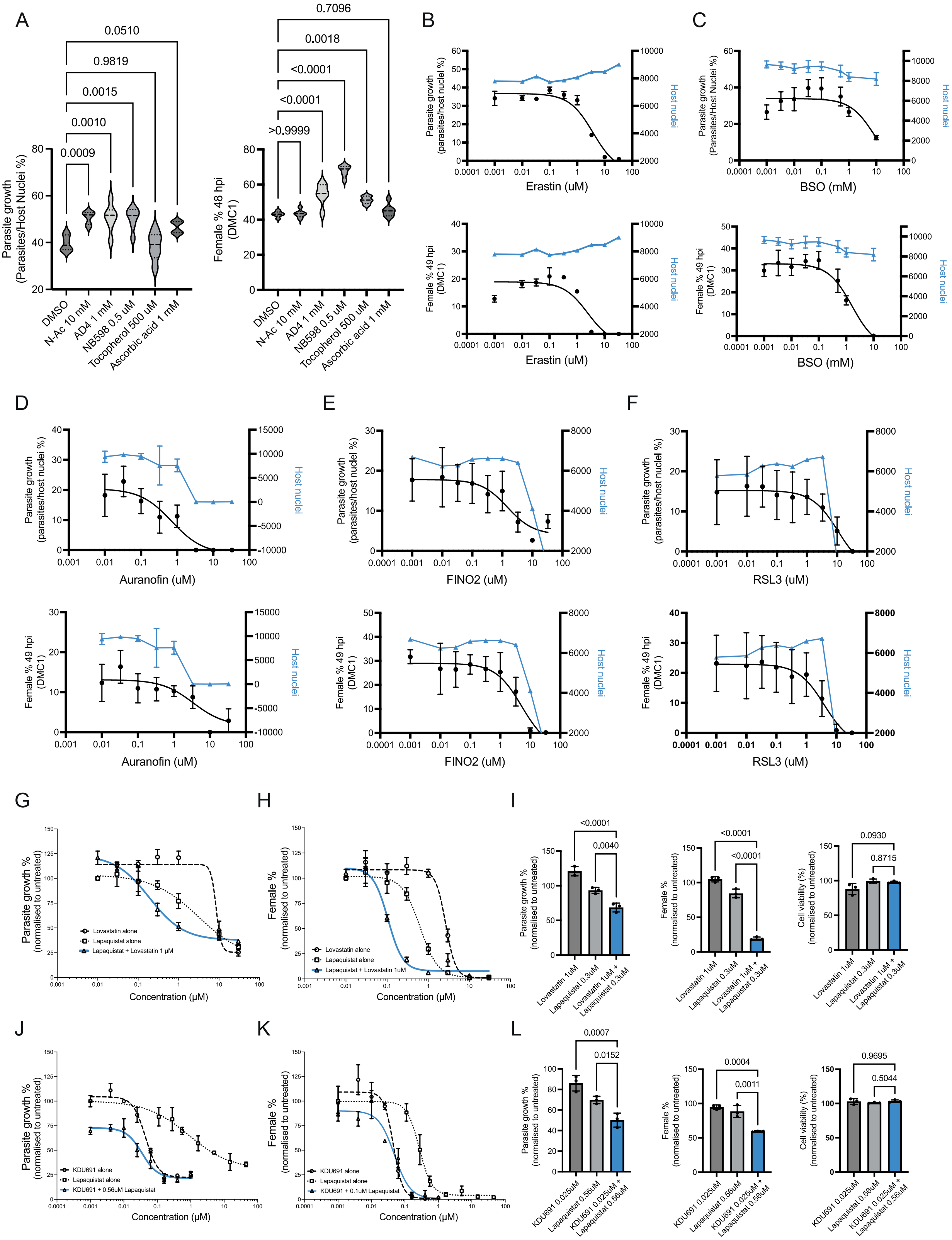

(legend on next page)

Figure S5. *Cryptosporidium* growth *in vitro* is affected by pro- and antioxidants, related to Figures 4 and 5

(A) Parasite growth (left) and female development (right) in HCT-8 cells in the presence of various antioxidants: N-Ac (10 mM), AD4 (1 mM), NB-598 (0.5 μM), tocopherol (500 μM), and ascorbic acid (1 mM). Violin plots depict the median value at the heavy dashed line and the lower and upper quartiles at the lighter dashed lines for 6 culture wells per condition. *p* values calculated by one-way ANOVA with Dunnett's multiple comparisons test.

(B) Erastin reduces parasite growth (top) and female development (bottom) in a dose-dependent manner with a minimal effect on host cell viability (blue lines). Parasite data are also shown in Figure 4C. Mean and SD for 3 culture wells shown, representative of three independent experiments.

(C) BSO reduces parasite growth (top) and female development (bottom) in a dose-dependent manner with a minimal effect on host cell viability (blue line). Mean and SD for 3 culture wells per condition shown, representative of two independent experiments.

(D) Auranofin reduces parasite growth (top) and female development (bottom) while also causing host cell death in a dose-dependent manner (blue lines). Mean and SD for 6 culture wells per condition shown, representative of two independent experiments.

(E and F) (E) FINO2 and RSL3 (F) reduce parasite growth (top) and female development (bottom) while also causing host cell death in a dose-dependent manner (blue lines). Mean and SD for 6 culture wells per condition shown.

(G and H) (G) Dose-response curves for the effect of lapaquistat or lovastatin alone, or (H) in combination on *Cryptosporidium* growth or female development in HCT-8 cells. Mean and SD for 3 culture wells per condition shown.

(I) Specific combinations of lapaquistat and lovastatin at indicated drug concentrations showing synergistic effects on parasite growth and female development are shown, as well as their minimal effects on host cell viability at the same concentrations. Combination index (CI) values of 0.2 and 0.1 for 50% inhibition of parasite growth and female development, respectively, indicating synergistic effects of the two drug combinations.[74] Loewe synergy scores of 3.3 ± 2.2 for parasite growth and 3.2 ± 1.6 for female development.[75]

(J and K) (J) Dose-response curves for the effect of lapaquistat or KDU691, a *Cryptosporidium* PI4K inhibitor[76] alone, or (K) in combination on parasite growth or female development in HCT-8 cells. Mean and SD for 3 culture wells per condition shown.

(L) Specific combinations of lapaquistat and KDU691 at indicated drug concentrations showing additive inhibitory effects on parasite growth and female development are shown, as well as their minimal effects on host cell viability at the same concentrations. CI values of 0.95 and 1.2 for 50% inhibition of parasite growth and female development, respectively, indicating additive effects of the two drug combinations. Loewe synergy scores of −3.9 ± 1.4 for parasite growth and −3.4 ± 3.6 for female development. *p* values calculated by one-way ANOVA with Dunnett's multiple comparisons test.
